# Sedentary Behaviors, Light-Intensity Physical Activity, and Healthy Aging

**DOI:** 10.1001/jamanetworkopen.2024.16300

**Published:** 2024-06-11

**Authors:** Hongying Shi, Frank B. Hu, Tianyi Huang, Eva S. Schernhammer, Walter C. Willett, Qi Sun, Molin Wang

**Affiliations:** 1 Department of Epidemiology and Health Statistics, School of Public Health, Wenzhou Medical University, Zhejiang, China; 2 Department of Epidemiology, Harvard T.H. Chan School of Public Health, Boston, Massachusetts; 3 Department of Nutrition, Harvard T.H. Chan School of Public Health, Boston, Massachusetts; 4 Channing Division of Network Medicine, Department of Medicine, Brigham and Women’s Hospital, Boston, Massachusetts; 5 Department of Epidemiology, Center for Public Health, Medical University of Vienna, Vienna, Austria; 6 Department of Biostatistics, Harvard T.H. Chan School of Public Health, Boston, Massachusetts

## Abstract

**Importance:**

Sleep duration and moderate-to-vigorous physical activity (MVPA) are associated with healthy aging, but the associations of sedentary behaviors and light-intensity physical activity (LPA) with healthy aging are still unclear.

**Objective:**

To examine the independent association of sedentary behaviors and LPA with healthy aging, and to estimate the theoretical association of replacing sedentary behavior with LPA, MVPA, or sleep with healthy aging.

**Design, Setting, and Participants:**

In this cohort study using data from the Nurses’ Health Study, participants aged 50 years or older and free of major chronic diseases in 1992 were prospectively followed up for 20 years. Data were analyzed from January to May 2022.

**Exposures:**

Three measures for sedentary behaviors (hours watching television, sitting at work, and other sitting at home) and 2 measures for LPA (hours of standing or walking around at home [LPA-Home] and at work [LPA-Work]).

**Main Outcomes and Measures:**

Healthy aging was defined as survival to at least age 70 years with maintenance of 4 health domains (ie, no major chronic diseases and no impairment in subjective memory, physical function, or mental health). The isotemporal substitution model was used to evaluate the potential impact on healthy aging of replacing 1 hour of 1 behavior with equivalent duration of another.

**Results:**

Among 45 176 participants (mean [SD] age, 59.2 [6.0] years), 3873 (8.6%) women achieved healthy aging. After adjustment for covariates including MVPA, each increment of 2 hours per day in sitting watching television was associated with a 12% (95% CI, 7%-17%) reduction in the odds of healthy aging. In contrast, each increase of 2 hours per day in LPA-Work was associated with a 6% (95% CI, 3%-9%) increase in the odds of healthy aging. Replacing 1 hour of sitting watching television with LPA-Home (OR, 1.08; 95% CI, 1.05-1.12), LPA-Work (OR, 1.10; 95% CI, 1.07-1.14), or MVPA (OR, 1.28; 95% CI, 1.23-1.34) was associated with increased odds of healthy aging. Among participants who slept 7 hours per day or less, replacing television time with sleep was also associated with increased odds of healthy aging.

**Conclusions and Relevance:**

In this cohort study, longer television watching time decreased odds of healthy aging, whereas LPA and MVPA increased odds of healthy aging and replacing sitting watching television with LPA or MVPA, or with sleep in those who slept 7 hours per day or less, was associated with increased odds of healthy aging, providing evidence for rearranging 24-hour behavior to promote overall health.

## Key Points

**Question:**

Besides moderate to vigorous physical activity and sleep, are sedentary behavior and light physical activity independently associated with healthy aging, and how could they be reallocated to promote healthy aging?

**Findings:**

In this cohort study among 45 176 female participants in the Nurses’ Health Study, sedentary behavior was associated with reduced odds of healthy aging, while light physical activity was associated with increased odds of healthy aging. Replacing television time with light physical activity, moderate to vigorous physical activity, or sleep (in participants with inadequate sleep) were associated with better odds of healthy aging.

**Meaning:**

These findings expand on the literature reporting that replacing sedentary behavior with light or moderate to vigorous physical activity is associated with decreased mortality by suggesting that this increased lifespan might be accompanied by better overall health.

## Teaser

This cohort study examines independent associations of sedentary behaviors, particularly watching television, and light physical activity with healthy aging and estimates the potential associations of replacing sedentary behaviors with physical activity or sleep.

## Introduction

Population aging is an important public health issue: 8.5% of the total population worldwide is aged 65 years and older, and this number is expected to increase to 20% by 2050.^1^ Aging is usually accompanied by adverse health conditions, including chronic diseases, cognitive decline, psychological disorders, and physical function limitations, causing a huge burden on individuals, families, and society.^2^ Approaches to achieve healthy aging, typically defined as being disease-free and physically, mentally, and cognitively healthy,^3,4^ are urgently needed. However, only 10% to 35% older adults achieve healthy aging.^5,6^ Identifying modifiable factors for healthy aging can inform interventions to promote this outcome.

Twenty-four–hour behaviors can be divided into sleep behavior, sedentary behavior (SB), light-intensity physical activity (LPA), and moderate- to vigorous-intensity physical activity (MVPA), which are important modifiable factors for health. Among them, MVPA has been associated with increased odds of healthy aging^7^ and sleep duration has been shown to have an inverted U-shaped association with healthy aging, with 7 hours daily sleep associated with the highest odds of healthy aging^4^; however, as potentially important behavioral intervention targets for older adults,^8,9,10,11,12^ the associations among SB, LPA, and healthy aging are rarely reported.^13^ In modern society, MVPA accounts for only approximately 4% of adults’ waking time, whereas duration spent on SB takes up approximately 60% of adults’ waking time and has significantly increased over time.^14,15^ Exploring the association of SB with the health of older adults has become particularly important. Using isotemporal substitution modeling (ISM), studies have found that replacing SB with physical activity could reduce the risk of mortality.^16,17^ However, it remains unclear whether the same substitutions can promote healthy aging, which considers not only survival status but also disease status and mental, physical, and cognitive function.

In this prospective cohort study, we aimed to evaluate the independent associations of different types of SBs and LPAs with healthy aging, and to examine the theoretical substitution association of replacing SBs with LPA, MVPA, and sleep in the Nurses’ Health Study (NHS), which will provide evidence for rationally arranging 24-hour behaviors and promoting the health of the elderly.

## Methods

The protocol for this cohort was approved by the institutional review boards of the Brigham and Women’s Hospital and the Harvard T.H. Chan School of Public Health. Participants provided implied consent by returning the questionnaires. This study followed the Strengthening the Reporting of Observational Studies in Epidemiology (STROBE) reporting guideline.

### Study Participants

The NHS was established in 1976, and information on lifestyle and health outcomes has been updated biennially. For this analysis, study baseline was defined as 1992, when information on time spent on SBs was first collected.^18^ Participants aged 50 years or older and free of major chronic diseases were potentially eligible and followed aup for 20 years, when all participants could potentially reach age 70 years (eFigure 1 in Supplement 1). We excluded individuals with missing information on time sitting watching television, implausible values on exposure variables,^19,20,21^ or missing assessment of healthy aging, leaving 45 176 women in the final analysis. Participants with missing healthy aging status did not differ substantially from those with this information (eTable 1 in Supplement 1).

### Assessment of Behaviors

We included 3 SBs, 2 LPAs, MVPA, and sleep duration (eFigure 2 in Supplement 1). Of these, we considered time spent sitting watching television as our primary exposure, because among the various surrogates for SB, time spent television watching is most strongly associated with adverse health outcomes.^18,22^ This variable was assessed by the question “On average, how many HOURS PER WEEK do you spend: sitting at home while watching TV [television] /VCR [videocassette recorder]?” SB-Work was assessed by “On average, how many HOURS PER WEEK do you spend: sitting at work or away from home or while driving,” which mainly reflect time spent sitting at work. SB-Home was assessed by “On average, how many HOURS PER WEEK do you spend: other sitting at home (e.g., reading, meal times, at desk)?” which does not include television watching. LPA-Work was assessed by “On average, how many HOURS PER WEEK do you spend: standing or walking around at work or away from home?” mainly reflecting occupational activity. LPA-Home was assessed by “On average, how many HOURS PER WEEK do you spend: standing or walking around at home?” typically reflecting household work. The 9 possible responses for each behavior ranged from 0 hours per week to more than 90 hours per week.

MVPA was measured by asking participants how much time they spent on 9 different recreational activities (walking; jogging; running; bicycling; tennis, squash, or racquetball; lap swimming; calisthenics, aerobics, aerobic dance, or rowing machine; yoga, stretching, or toning; and lawn mowing).^23,24^ The response for each activity had 10 categories, ranging from 0 minutes per week to 11 or more hours per week. Participants also reported their usual walking pace in miles per hour (mph): easy (<2 mph), average (2-2.9 mph), brisk (3-3.9 mph), very brisk (≥4 mph), or unable to walk; and the mean number of flights of stairs they climbed daily. Based on this information and intensity of each activity, expressed by metabolic equivalent task (MET), we calculated total weekly MET-hours.^25^ We defined activities requiring at least 6 METs per hour as vigorous-intensity activities^25,26^; and walking (2-4.5 METs, depending on pace), was considered as moderate-intensity activity.^25^ These 2 variables were combined as MVPA. Sleep duration was assessed by asking respondents how long they usually sleep in a 24-hour cycle (options were ≤5, 6, 7, 8, 9, 10, and ≥11 hours).^4^

The reliability and validity of self-administered questionnaires on these behaviors were examined previously.^24,27^ Specifically, correlations between physical inactivity reported in diaries and reported on questionnaires were reasonable (*r* = 0.41 to *r* = 0.44); correlation between self-administered questionnaires with true MVPA was *r* = 0.60; and test-retest coefficients were *r* = 0.52 to *r* = 0.55.^24,27^

### Assessment of Healthy Aging

To comprehensively assess the health status of the respondents and based on the concept of successful aging proposed by Rowe and Kahn^28^ and other related studies,^4,29,30,31^ we defined healthy aging as surviving to at least age 70 years with maintenance of 4 health domains, including being free of 11 main chronic diseases and no impairment of physical function, memory, or mental health. Participants who did not meet these 4 domains or died during the 20 years of follow-up were classified as usual agers.^32^ The specific evaluation methods and judgment criteria for the 4 dimensions are shown in eAppendix 1 in Supplement 1.

### Statistical Analysis

To evaluate the independent associations of SBs and LPAs with healthy aging, we adjusted for age; education; marital status; annual household income; family history of cancer, myocardial infarction, and diabetes; baseline hypertension and high cholesterol; menopausal status and postmenopausal hormone use; aspirin use; smoking history, alcohol intake, total energy intake, and diet quality (assessed by a validated semiquantitative food frequency questionnaire)^33,34,35^; and sleep duration. Considering that body mass index (BMI) could be in the causal pathway between 24-hour behaviors and healthy aging, we ran an additional model further adjusting for BMI. Considering the importance of age on the evaluation of healthy aging, we examined the association between television time and healthy aging stratified by age. Moreover, we calculated the population-attributable risk, an estimate of the percentage of individuals with healthy aging during follow-up that could have been achieved if they engaged in the low-risk category for these behaviors, assuming that the observed associations were causal.^36^

Then to quantify the associations of replacing 1 hour of a behavior for equal amount of another behavior with healthy aging while the total amount of time of all behaviors was kept constant, we fitted the ISM (and a partition model for comparison) (eAppendix 2 in Supplement 1). We modeled sleep as a piecewise variable with a cutoff at 7 hours (≤7 hours per day and >7 hours per day)^19,20^ to account for the inverted U-shaped association between sleep duration and healthy aging.^4^ Because the proportion of missing values for each exposure was less than 5% (the highest was only 3.1% for SB-Work), imputation using the median values was used in the main ISM analysis. In addition, we performed the complete cases analysis. To further verify the robustness of our results, for women who had at least 1 of the time use variables missing, we used multiple imputations (10 imputations) and the expectation-maximization algorithm.^19,20^

Similarly, we analyzed the independent and replacement association of all exposures with the 4 domains of healthy aging. Potential heterogeneity in the association was also explored by stratified analysis by MVPA (being physically active or inactive, using a threshold of 7.5 MET hours per week, corresponding to the minimum physical activity recommendations^37^). In secondary analyses, to further evaluate the association between these exposures and healthy aging among survivors, we excluded participants who died before 2012 from usual agers, and then repeated all analyses.

All statistical tests were 2-sided, and *P* < .05 was considered statistically significant. Data management and statistical analyses were performed using SAS software version 9.4 (SAS Institute). Data were analyzed from January to May 2022.

## Results

In total, 45 176 women were included (mean [SD] age 59.2 [6.0] years; range, 50-72 years). After 20 years of follow-up, 3873 women (8.6%) achieved healthy aging (by age at baseline: 50 years, 18.2%; 55 years, 9.2%; 60 years, 4.1%; 65 years, 0.9%). As for the 4 domains of healthy aging, 18 696 women (41.4%) had none of the 11 chronic diseases, 7250 women (16.1%) had no impairment of physical function, 19 937 women (44.1%) had no mental health limitation, and 23 465 women (51.9%) reported no impairment of memory function.

### Baseline Characteristics of Participants

Participants with longer television watching time were older, less educated, more likely to smoke or drink alcohol, more likely to have hypertension and high cholesterol, and more likely to have higher BMI and calorie intake and lower diet quality, compared with those who spent less time watching television (Table 1). Moreover, time spent watching television was not correlated with MVPA (MET-hours/week) (Spearman correlation coefficient, −0.026), indicating that television watching and MVPA were independent behaviors.

**Table 1.** Age-Adjusted Baseline Characteristics by Time Spent Watching Television in Women in the Nurses’ Health Study^a^

Characteristics	Participants by television h/wk, No. (%) (N = 45 176)				
	0-1 (n = 3020)	2-5 (n = 10 815)	6-20 (n = 23 738)	21-40 (n = 6594)	≥41 (n = 1009)
Age, mean (SD), y^b^	57.6 (5.7)	58.6 (6.0)	59.1 (5.9)	60.9 (5.9)	61.8 (5.8)
Education					
Registered nurse	1881 (62.6)	7268 (67.5)	16 648 (70.4)	4980 (75.9)	758 (75.5)
Bachelor’s degree	680 (22.6)	2252 (20.9)	4708 (19.9)	1168 (17.8)	176 (17.6)
Master’s or doctorate degree	446 (14.8)	1241 (11.5)	2284 (9.7)	418 (6.4)	69 (6.9)
Husband’s education					
≤High school	990 (41.2)	3835 (42.9)	9162 (45.4)	2906 (50.6)	436 (50.7)
College graduate	687 (28.6)	2735 (30.6)	5995 (29.7)	1649 (28.7)	247 (28.8)
Graduate school	724 (30.2)	2360 (26.4)	5028 (24.9)	1187 (20.7)	176 (20.5)
Marital status					
Married	2336 (77.4)	8722 (80.8)	19 578 (82.6)	5532 (84.1)	778 (77.1)
Widowed	336 (11.2)	1157 (10.7)	2384 (10.1)	639 (9.7)	141 (14.0)
Separated, divorced, or never married	344 (11.4)	912 (8.5)	1746 (7.4)	410 (6.2)	90 (8.9)
Family annual income, median (IQR), $10 000	6.1 (4.7-8.0)	6.0 (4.7-7.8)	6.0 (4.7-7.8)	5.9 (4.7-7.5)	5.7 (4.5-7.3)
BMI	24.9 (4.5)	25.4 (4.6)	25.8 (4.7)	26.7 (5.2)	27.3 (6.1)
Smoking status					
Never	1570 (52.0)	4979 (46.0)	10 349 (43.6)	2597 (39.4)	385 (38.2)
Past	1069 (35.4)	4369 (40.4)	9968 (42.0)	2863 (43.4)	428 (42.4)
Current	381 (12.6)	1467 (13.6)	3421 (14.4)	1134 (17.2)	196 (19.4)
Alcohol intake, g/d					
None	1454 (51.2)	4791 (46.7)	9847 (43.4)	2759 (43.7)	463 (48.3)
1-14.9	1134 (39.9)	4491 (43.8)	10 515 (46.3)	2827 (44.7)	380 (39.6)
≥15	254 (8.9)	972 (9.5)	2343 (10.3)	734 (11.6)	116 (12.1)
Diet quality^c^	50.7 (11.1)	49.4 (10.4)	48.1 (10.3)	46.4 (10.6)	45.9 (10.6)
Total energy intake, mean (SD), kcal/d	1702.5 (526.5)	1701.8 (517.7)	1749.5 (514.0)	1798.0 (525.8)	1790.5 (539.4)
Use of multivitamin	1362 (45.5)	4770 (44.5)	10043 (42.7)	2742 (41.9)	411 (41.1)
Regular aspirin (≥2 tabs/wk)	797 (26.4)	2901 (26.8)	6914 (29.1)	1980 (30.0)	290 (28.7)
Family history of diabetes	773 (25.6)	3049 (28.2)	6731 (28.4)	1904 (28.9)	289 (28.6)
Family history of myocardial infarction	507 (16.8)	1946 (18.0)	4401 (18.5)	1264 (19.2)	213 (21.1)
Family history of cancer	404 (13.4)	1467 (13.6)	3291 (13.9)	898 (13.6)	159 (15.8)
Hypertension	572 (18.9)	2350 (21.7)	5397 (22.7)	1664 (25.2)	297 (29.5)
High cholesterol	872 (28.9)	3290 (30.4)	7933 (33.4)	2445 (37.1)	389 (38.5)
Menopausal status and hormone use					
Premenopausal	308 (10.4)	1019 (9.6)	2178 (9.3)	616 (9.5)	63 (6.3)
Postmenopausal and never used hormones	1447 (48.8)	5120 (48.5)	11 158 (47.8)	3141 (48.5)	515 (51.8)
Postmenopausal and past hormone user	98 (3.3)	342 (3.2)	770 (3.3)	206 (3.2)	27 (2.7)
Postmenopausal and current hormone user	1112 (37.5)	4083 (38.6)	9259 (39.6)	2516 (38.8)	389 (39.2)
Activities in 24 h, median (IQR), h/d					
Television time	0.1 (0.1-0.1)	0.5 (0.5-0.5)	2.2 (1.1-2.2)	4.4 (4.4-4.4)	7.2 (7.2-10.8)
SB-Work	0.5 (0.5-2.2)	0.5 (0.5-2.2)	1.1 (0.5-2.2)	1.1 (0.5-2.2)	1.1 (0.5-4.4)
SB-Home	1.1 (0.5-2.2)	0.5 (0.5-1.1)	1.1 (1.1-2.2)	2.2 (1.1-4.4)	4.4 (1.1-7.2)
LPA-Home	2.2 (1.1-4.4)	2.2 (1.1-4.4)	2.2 (1.1-4.4)	2.2 (1.1-4.4)	4.4 (1.1-4.4)
LPA-Work	1.1 (0.5-4.4)	2.2 (0.5-4.4)	2.2 (0.5-4.4)	1.1 (0.5-4.4)	1.1 (0.5-4.4)
Standardized MVPA	0.8 (0.3-1.8)	0.8 (0.3-1.8)	0.8 (0.3-1.7)	0.7 (0.3-1.6)	0.7 (0.2-1.6)
Sleep duration	6.9 (1.0)	6.9 (1.0)	7.0 (0.9)	7.1 (1.0)	7.0 (1.1)

Abbreviations: BMI, body mass index (calculated as weight in kilograms divided by the square of height in meters); LPA-Home, standing or walking around at home; LPA-Work, standing or walking around at work; MVPA, moderate-to-vigorous physical activity; SB-Home, other sitting at home; SB-Work, sitting at work or away from home or driving.

^a^ Values are standardized to the age distribution of the study population.

^b^ Value was not age adjusted.

^c^ Measured as Alternate-Healthy Eating Index (range, 0-100; higher score indicates better diet quality).

### Independent Associations Among SB, LPA, and Healthy Aging

Sitting time spent watching television was inversely associated with odds of healthy aging (Table 2). Compared with women who watched television for 1 hour per week or less, increasing time spent watching television was associated with decreasing odds of healthy aging (2-5 hours per week: OR, 0.91; 95% CI, 0.80-1.04; 6-20 hours per week: OR, 0.81; 95% CI, 0.72-0.92; 21-40 hours per week: OR, 0.60; 95% CI, 0.51-0.71; and ≥41 hours per week: OR, 0.55; 95% CI, 0.38-0.79; *P *for trend < .001). These associations were attenuated after further adjustment for BMI. In multivariate-adjusted model, SB-Work (2-5 hours per week: OR, 1.05; 95% CI, 0.89-1.23; 6-20 hours per week: OR, 0.97; 95% CI, 0.83-1.14; 21-40 hours per week: OR, 0.93; 95% CI, 0.78-1.11; ≥41 hours per week: OR, 0.89; 95% CI, 0.70-1.13; *P* for trend = .03) and SB-Home (2-5 hours per week: OR, 1.22; 95% CI, 0.99-1.50; 6-20 hours per week: OR, 1.12; 95% CI, 0.92-1.37; 21-40 hours per week: OR, 1.01; 95% CI, 0.81-1.27; ≥41 hours per week: OR, 1.01; 95% CI, 0.72-1.41; *P* for trend = .01) were also significantly associated with reduced odds of healthy aging, but these associations were attenuated after further adjustment for BMI, and SB-Work was no longer significant.

**Table 2.** Odds of Healthy Aging by Sedentary Behaviors and Light-Intensity Physical Activity

Exposures, per wk	OR (95% CI) by h/wk of behavior					*P* value for trend	Per 2 h/d, OR (95% CI)^a^
	0-1	2-5	6-20	21-40	≥41		
**Sitting while watching television (n = 45 176)**							
Healthy ager, No. (%)	401 (13.3)	1131 (10.5)	2010 (8.5)	297 (4.5)	34 (3.4)	NA	NA
Age-adjusted	1 [Reference]	0.85 (0.75-0.97)	0.72 (0.64-0.81)	0.47 (0.40-0.55)	0.39 (0.27-0.57)	<.001	0.77 (0.72-0.81)
Multivariable adjusted^b^	1 [Reference]	0.91 (0.80-1.04)	0.81 (0.72-0.92)	0.60 (0.51-0.71)	0.55 (0.38-0.79)	<.001	0.84 (0.79-0.89)
Multivariable and BMI adjusted^c^	1 [Reference]	0.95 (0.83-1.08)	0.87 (0.77-0.99)	0.68 (0.58-0.81)	0.61 (0.42-0.89)	<.001	0.88 (0.83-0.93)
**Sitting at work or away from home or driving (n = 43 769)**							
Healthy ager, No. (%)	206 (5.6)	1056 (7.6)	1812 (9.3)	585 (10.9)	134 (10.2)	NA	NA
Age-adjusted	1 [Reference]	1.12 (0.96-1.32)	1.06 (0.91-1.24)	1.06 (0.89-1.25)	1.00 (0.79-1.27)	.33	1.01 (0.97-1.05)
Multivariable adjusted^b^	1 [Reference]	1.05 (0.89-1.23)	0.97 (0.83-1.14)	0.93 (0.78-1.11)	0.89 (0.70-1.13)	.03	0.97 (0.93-1.01)
Multivariable and BMI adjusted^c^	1 [Reference]	1.04 (0.88-1.22)	0.98 (0.84-1.15)	0.95 (0.80-1.14)	0.94 (0.74-1.20)	.16	0.98 (0.94-1.03)
**Other sitting at home (n = 44 852)**							
Healthy ager, No. (%)	121 (7.6)	1094 (9.7)	2229 (8.8)	347 (6.4)	61 (5.3)	NA	NA
Age-adjusted	1 [Reference]	1.30 (1.06-1.59)	1.21 (0.99-1.48)	1.10 (0.88-1.37)	1.06 (0.76-1.47)	.032	1.01 (0.96-1.06)
Multivariable adjusted^b^	1 [Reference]	1.22 (0.99-1.50)	1.12 (0.92-1.37)	1.01 (0.81-1.27)	1.01 (0.72-1.41)	.01	0.99 (0.94-1.05)
Multivariable and BMI adjusted^c^	1 [Reference]	1.23 (0.99-1.51)	1.13 (0.93-1.39)	1.02 (0.81-1.29)	1.05 (0.75-1.48)	.023	0.99 (0.94-1.05)
**Standing or walking around at home (n = 44 519)**							
Healthy ager, No. (%)	45 (5.5)	451 (7.3)	1725 (9.0)	1030 (9.0)	559 (8.2)	NA	NA
Age-adjusted	1 [Reference]	1.38 (1.00-1.91)	1.61 (1.18-2.20)	1.74 (1.27-2.38)	1.86 (1.35-2.57)	<.001	1.03 (1.01-1.06)
Multivariable adjusted^b^	1 [Reference]	1.33 (0.96-1.85)	1.46 (1.06-2.01)	1.52 (1.10-2.10)	1.59 (1.14-2.20)	.005	1.04 (1.01-1.07)
Multivariable and BMI adjusted^c^	1 [Reference]	1.35 (0.96-1.88)	1.45 (1.05-2.00)	1.46 (1.05-2.02)	1.50 (1.08-2.09)	.11	1.02 (0.99-1.05)
**Standing or walking around at work (n = 43 838)**							
Healthy ager, No. (%)	137 (3.7)	558 (5.9)	1342 (8.6)	1258 (11.9)	523 (11.8)	NA	NA
Age-adjusted	1 [Reference]	1.24 (1.02-1.51)	1.39 (1.15-1.67)	1.53 (1.27-1.85)	1.45 (1.19-1.78)	<.001	1.02 (0.99-1.05)
Multivariable adjusted^b^	1 [Reference]	1.13 (0.93-1.38)	1.21 (1.00-1.46)	1.43 (1.19-1.74)	1.40 (1.14-1.72)	<.001	1.05 (1.02-1.08)
Multivariable and BMI adjusted^c^	1 [Reference]	1.13 (0.92-1.38)	1.21 (1.00-1.47)	1.42 (1.17-1.72)	1.42 (1.16-1.75)	<.001	1.06 (1.03-1.09)
**Standardized MVPA (n = 45 176)** ^d^							
Healthy ager, No. (%)	435 (4.6)	402 (7.0)	800 (8.3)	1092 (10.1)	1144 (12.2)	NA	NA
Age adjusted	1 [Reference]	1.52 (1.31-1.75)	1.87 (1.65-2.11)	2.47 (2.20-2.78)	3.18 (2.83-3.58)	<.001	1.20 (1.18-1.23)^e^
Multivariable adjusted^b^	1 [Reference]	1.41 (1.22-1.63)	1.68 (1.48-1.90)	2.13 (1.89-2.40)	2.69 (2.38-3.03)	<.001	1.18 (1.15-1.20)^e^
Multivariable and BMI adjusted^c^	1 [Reference]	1.31 (1.13-1.52)	1.51 (1.33-1.72)	1.85 (1.64-2.09)	2.20 (1.94-2.49)	<.001	1.14 (1.11-1.16)^e^

Abbreviations: BMI, body mass index (calculated as weight in kilograms divided by height in meters squared); MVPA, moderate to vigorous physical activity; NA, not applicable; OR, odds ratio.

^a^ All sedentary behavior, light-intensity physical activity variables, and standardized MVPA are included simultaneously in this model. Standardized MVPA (hours per day) is the total metabolic equivalent task-hours per week / 3 (for 1 hour of normal-pace walking) / 5 days per week. The sample size for this model is 42 368.

^b^ Adjusted for age (years); education (registered nurse, bachelor’s, or graduate); marital status (married, widowed, or separated or divorced); household income (quintiles); family history of cancer, myocardial infarction, and diabetes (yes or no); baseline hypertension and high cholesterol (yes or no); menopausal status and postmenopausal hormone use (premenopausal, postmenopausal and never user, postmenopausal and past user, postmenopausal and current user), aspirin use (regular use or not); smoking history (never, former, current), alcohol intake (none, 1-14.9, ≥15 g/d), total energy intake (quintiles), diet quality (Alternate Healthy Eating Index score, quintiles), sleep duration (≤5, 6, 7, 8, ≥9 hours per day), MVPA (MET-h/wk, in quintiles) (except for MVPA).

^c^ Categorized as <18.5, 18.5-24.9, 25-29.9, or 30 or greater.

^d^ The categories for MVPA were less than 15 minutes per day, 15 to 29 minutes per day, 30 to 59 minutes per day, 1 to 2 hours per day and 2 or more hours per day, with the category of less than 15 minutes per day as the reference group.

^e^ Assessed as per 1 hour of MVPA per day, regardless of sedentary behavior.

In contrast, time spent on LPAs was associated with higher odds of healthy aging. For LPA-Home, the multivariate-adjusted OR comparing 41 hours per week or more vs 0 to 1 hour per week was associated with 59% higher odds of health aging (OR, 1.59; 95% CI, 1.14-2.20; *P *for trend = .005); additional adjustment for BMI attenuated the association. Similarly, LPA-Work was associated with higher odds of healthy aging for participants who watched 41 hours of television per week or more compared with those who watched 1 hour or less (multivariate-adjusted OR, 1.40; 95% CI, 1.14-1.72; *P *for trend < .001); further adjustment for BMI did not appreciably change the results.

Stratified analysis by age groups showed that the association between time spent watching television and healthy aging among the older age group was stronger than that in the younger age group (*P* for interaction = .001) (eTable 2 in Supplement 1). Stratified analysis by MVPA showed that increasing MVPA did not completely negate the decreased odds of healthy aging associated with prolonged television watching (eFigure 3 in Supplement 1).

To compare the independent role associated with SBs, LPAs, and MVPA, we conducted a multivariate analysis including all these behaviors and potential confounders simultaneously (Table 2; eFigure 4 in Supplement 1). Time spent watching television was most strongly associated with odds of healthy aging. For each increase of 2 hours per day in time spent watching television, there was a 12% (95% CI, 7%-17%) decrease in odds of healthy aging. In contrast, LPA-Work was associated with significantly higher odds of healthy aging, 6% (95% CI, 3%-9%) for each increase of 2 hours per day. Additionally, each increase of 1 hour per day in standardized MVPA (normal-pace walking or equivalent energy expenditure) was associated with a 14% (95% CI, 11%-16%) improvement in odds of healthy aging. Associations for the remaining 3 behaviors were not statistically significant.

We estimated that 61% (95% CI, 53%-68%) of usual agers could become healthy agers if they adhered to 4 lifestyle factors: less than 3 hours per day of television watching, at least 3 hours per day of LPA-Work, and at least 30 minutes per day standardized MVPA, and no overweight or obesity (eTable 3 in Supplement 1). However, only 4835 participants (11.0%) in our cohort belonged to the joint low-risk group.

### Theoretical Replacement Outcomes of SB and LPA for Healthy Aging

In the ISM analyses (Table 3), total activities were broken down into 6 components (sitting watching television, SB-Work, SB-Home, LPA-Home, LPA-Work, and MVPA). Replacing sitting watching television with any activity (SB-Work, SB-Home, LPA-Home, LPA-Work, or MVPA) was associated with increased odds of healthy aging, and the increases were greater when it was replaced with higher intensities of physical activity. For example, replacing 1 hour per day of television watching with 1 hour per day of MVPA was associated with 28% higher odds of healthy aging (OR, 1.28; 95% CI, 1.23-1.34). Conversely, replacing MVPA with any other activity reduced the odds of healthy aging. In addition, replacing SB-Work with LPA-Work was associated with increased odds of healthy aging.

**Table 3.** Odds of Healthy Aging According to Isotemporal Substitution of 1 Hour per Day of 6 Activities

Analysis model	OR (95% CI)^a^					
	Sitting while watching television	SB-Work	SB-Home	LPA-Home	LPA-Work	Standardized MVPA^b^
Substitution model						
Replace television watching with	Replaced	1.07 (1.03-1.11)	1.07 (1.02-1.11)	1.08 (1.05-1.12)	1.10 (1.07-1.14)	1.28 (1.23-1.34)
Replace SB-Work with	0.94 (0.90-0.97)	Replaced	1.00 (0.96-1.04)	1.02 (0.99-1.04)	1.04 (1.01-1.06)	1.20 (1.16-1.25)
Replace SB-Home with	0.94 (0.90-0.98)	1.00 (0.96-1.04)	Replaced	1.02 (0.98-1.05)	1.03 (1.00-1.07)	1.20 (1.16-1.25)
Replace LPA-Home with	0.92 (0.89-0.95)	0.98 (0.96-1.01)	0.98 (0.95-1.02)	Replaced	1.02 (1.00-1.04)	1.18 (1.14-1.23)
Replace LPA-Work with	0.91 (0.88-0.94)	0.97 (0.94-0.99)	0.97 (0.94-0.99)	0.98 (0.96-1.00)	Replaced	1.16 (1.12-1.20)
Replace MVPA with	0.78 (0.75-0.81)	0.83 (0.80-0.86)	0.83 (0.80-0.87)	0.84 (0.82-0.87)	0.86 (0.83-0.89)	Replaced
Partition model^c^	0.93 (0.91-0.96)	1.00 (0.97-1.02)	1.00 (0.97-1.02)	1.01 (1.00-1.03)	1.03 (1.02-1.05)	1.20 (1.16-1.24)

Abbreviations: LPA-Home, standing or walking around at home; LPA-Work, standing or walking around at work; MVPA, moderate-to-vigorous physical activity; OR, odds ratio; SB-Home, other sitting at home; SB-Work, sitting at work or away from home or driving.

^a^ Total time is the sum of time spent on sitting watching television, SB-Work, SB-Home, LPA-Home, LPA-Work, and MVPA. ORs are adjusted for age (years); education (registered nurse, bachelor, or graduate); marital status (married, widowed, or separated or divorced); household income (quintiles); family history of cancer, myocardial infarction, and diabetes (yes or no); baseline hypertension and high cholesterol (yes or no); menopausal status and postmenopausal hormone use (premenopausal, postmenopausal and never user, postmenopausal and past user, postmenopausal and current user); aspirin use (regular use or not); smoking history (never, former, current), alcohol intake (none, 1-14.9, ≥15 g/d), total energy intake (quintiles), diet quality (Alternate Healthy Eating Index score, quintiles), sleep duration (≤5, 6, 7, 8, ≥9 hours per day), and body mass index (calculated as weight in kilograms divided by height in meters squared and categorized as <18.5, 18.5-24.9, 25-29.9, ≥30).

^b^ Standardized as normal-pace walking time (hours per day).

^c^ Each OR represents a comparison of healthy aging for every 1 hour per day increase in the exposure variable, not restricting total time nor controlling the displacement of other activity time.

Stratification analysis by physical activity (Table 4) found that the apparent benefit of replacing any other activities with MVPA was greater among physically inactive participants. For example, replacing 1 hour of television watching with 1 hour of MVPA was associated with much higher odds of healthy aging among the physically inactive group (OR, 4.26; 95% CI, 2.00-9.06) than among the physically active group (OR, 1.21; 95% CI, 1.16-1.27).

**Table 4.** Odds of Healthy Aging According to Isotemporal Substitution of 1 Hour per Day of 6 Activities Stratified by Physical Activity

Model	OR (95% CI)^a^					
	Sitting while watching television	SB-Work	SB-Home	LPA-Home	LPA-Work	Standardized MVPA^b^
**Physically active participants (n = 29 939)**						
Substitution model						
Replace television watching with	Replaced	1.06 (1.01-1.10)	1.07 (1.02-1.13)	1.08 (1.04-1.12)	1.10 (1.06-1.14)	1.21 (1.16-1.27)
Replace SB-Work with	0.94 (0.91-0.99)	Replaced	1.01 (0.97-1.06)	1.02 (0.99-1.05)	1.04 (1.01-1.07)	1.15 (1.10-1.20)
Replace SB-Home with	0.93 (0.89-0.98)	0.99 (0.95-1.03)	Replaced	1.01 (0.97-1.04)	1.03 (0.99-1.06)	1.13 (1.08-1.19)
Replace LPA-Home with	0.92 (0.89-0.96)	0.98 (0.95-1.01)	0.99 (0.96-1.03)	Replaced	1.02 (0.99-1.04)	1.12 (1.08-1.17)
Replace LPA-Work with	0.91 (0.88-0.94)	0.96 (0.93-0.99)	0.97 (0.94-1.01)	0.98 (0.96-1.01)	Replaced	1.10 (1.06-1.15)
Replace MVPA with	0.82 (0.79-0.86)	0.87 (0.84-0.91)	0.88 (0.84-0.92)	0.89 (0.86-0.93)	0.91 (0.87-0.94)	Replaced
Partition model^c^	0.93 (0.90-0.97)	0.99 (0.97-1.01)	1.00 (0.97-1.03)	1.01 (0.99-1.03)	1.03 (1.01-1.05)	1.14 (1.10-1.17)
**Physically inactive participants (n = 15 237)**						
Substitution model						
Replace television watching with	Replaced	1.09 (1.01-1.18)	1.03 (0.94-1.13)	1.08 (1.01-1.16)	1.12 (1.05-1.20)	4.26 (2.00-9.06)
Replace SB-Work with	0.92 (0.85-0.99)	Replaced	0.95 (0.87-1.03)	0.99 (0.94-1.04)	1.03 (0.98-1.09)	3.91 (1.83-8.32)
Replace SB-Home with	0.97 (0.88-1.07)	1.06 (0.97-1.15)	Replaced	1.05 (0.97-1.13)	1.09 (1.02-1.17)	4.13 (1.94-8.81)
Replace LPA-Home with	0.93 (0.87-0.99)	1.01 (0.96-1.07)	0.96 (0.89-1.03)	Replaced	1.04 (0.99-1.10)	3.95 (1.85-8.40)
Replace LPA-Work with	0.89 (0.83-0.95)	0.97 (0.92-1.02)	0.92 (0.86-0.98)	0.96 (0.91-1.01)	Replaced	3.79 (1.78-8.05)
Replace MVPA with	0.24 (0.11-0.50)	0.26 (0.12-0.55)	0.24 (0.11-0.52)	0.25 (0.12-0.54)	0.26 (0.12-0.56)	Replaced
Partition model^c^	0.93 (0.88-0.99)	1.02 (0.97-1.06)	0.96 (0.90-1.02)	1.00 (0.97-1.04)	1.05 (1.02-1.08)	3.96 (1.87-8.43)

Abbreviations: LPA-Home, standing or walking around at home; LPA-Work, standing or walking around at work; MVPA, moderate-to-vigorous physical activity; OR, odds ratio; SB-Home, other sitting at home; SB-Work, sitting at work or away from home or driving.

^a^ Total time is the sum of time spent on sitting watching television, SB-Work, SB-Home, LPA-Home, LPA-Work, and MVPA. ORs are adjusted for age (years); education (registered nurse, bachelor, or graduate); marital status (married, widowed, or separated or divorced); household income (quintiles); family history of cancer, myocardial infarction, and diabetes (yes or no); baseline hypertension and high cholesterol (yes or no); menopausal status and postmenopausal hormone use (premenopausal, postmenopausal and never user, postmenopausal and past user, postmenopausal and current user); aspirin use (regular use or not); smoking history (never, former, current), alcohol intake (none, 1-14.9, ≥15 g/d), total energy intake (quintiles), diet quality (Alternate Healthy Eating Index score, quintiles), sleep duration (≤5, 6, 7, 8, ≥9 hours per day), and body mass index (calculated as weight in kilograms divided by height in meters squared and categorized as <18.5, 18.5-24.9, 25-29.9, ≥30).

^b^ Standardized as normal-pace walking time (hours per day).

^c^ Each OR represents a comparison of healthy aging for every 1 hour per day increase in the exposure variable, not restricting total time nor controlling the displacement of other activity time.

The above replacement outcomes were also observed in participants with different levels of sleep duration (Table 5). Of note, among individuals who slept 7 or fewer hours, the odds of healthy aging would be improved if time was reallocated into sleep only from television watching but not from any other activities.

**Table 5.** Odds of Healthy Aging According to Isotemporal Substitution of 1 Hour per Day of 7 Activities Stratified by Sleep Duration

Model	OR (95% CI)^a^						
	Sleep duration	Sitting while watching television	SB-Work	SB-Home	LPA-Home	LPA-Work	Standardized MVPA^b^
**Sleep ≤7 h per day (n = 27 535)**							
Substitution model							
Replace sleep with	Replaced	0.90 (0.83-0.98)	0.95 (0.88-1.02)	0.96 (0.88-1.03)	0.97 (0.90-1.04)	0.99 (0.92-1.06)	1.16 (1.07-1.25)
Replace television watching with	1.11 (1.02-1.20)	Replaced	1.05 (1.00-1.10)	1.06 (1.00-1.12)	1.07 (1.03-1.12)	1.10 (1.05-1.14)	1.28 (1.22-1.35)
Replace SB-Work with	1.05 (0.98-1.14)	0.95 (0.91-0.99)	Replaced	1.01 (0.96-1.05)	1.02 (0.99-1.06)	1.04 (1.01-1.08)	1.22 (1.17-1.28)
Replace SB-Home with	1.05 (0.97-1.13)	0.95 (0.89-1.00)	0.99 (0.95-1.04)	Replaced	1.02 (0.98-1.06)	1.04 (0.99-1.08)	1.21 (1.15-1.28)
Replace LPA-Home with	1.03 (0.96-1.11)	0.93 (0.89-0.97)	0.98 (0.95-1.01)	0.98 (0.94-1.03)	Replaced	1.02 (0.99-1.05)	1.19 (1.14-1.25)
Replace LPA-Work with	1.01 (0.94-1.09)	0.91 (0.88-0.95)	0.96 (0.93-0.99)	0.97 (0.93-1.00)	0.98 (0.95-1.01)	Replaced	1.17 (1.12-1.22)
Replace MVPA with	0.86 (0.80-0.93)	0.78 (0.74-0.82)	0.82 (0.78-0.86)	0.82 (0.78-0.87)	0.84 (0.80-0.88)	0.85 (0.82-0.89)	Replaced
Partition model^c^	1.04 (0.97-1.12)	0.94 (0.91-0.98)	0.99 (0.96-1.02)	0.99 (0.96-1.03)	1.01 (0.99-1.03)	1.03 (1.01-1.05)	1.21 (1.16-1.26)
**Sleep >7 h per day (n = 10 767)**							
Substitution model							
Replace sleep with	Replaced	1.11 (0.90-1.37)	1.21 (0.98-1.48)	1.15 (0.94-1.42)	1.18 (0.96-1.44)	1.22 (0.99-1.50)	1.40 (1.13-1.73)
Replace television watching with	0.90 (0.73-1.11)	Replaced	1.09 (1.01-1.17)	1.04 (0.95-1.13)	1.06 (0.99-1.14)	1.10 (1.03-1.18)	1.26 (1.16-1.38)
Replace SB-Work with	0.83 (0.68-1.02)	0.92 (0.85-0.99)	Replaced	0.96 (0.88-1.03)	0.98 (0.92-1.03)	1.01 (0.96-1.07)	1.16 (1.07-1.26)
Replace SB-Home with	0.87 (0.71-1.07)	0.96 (0.88-1.05)	1.05 (0.97-1.13)	Replaced	1.02 (0.96-1.09)	1.06 (0.99-1.13)	1.21 (1.12-1.32)
Replace LPA-Home with	0.85 (0.69-1.04)	0.94 (0.88-1.01)	1.02 (0.97-1.08)	0.98 (0.92-1.04)	Replaced	1.04 (0.99-1.09)	1.19 (1.10-1.28)
Replace LPA-Work with	0.82 (0.67-1.00)	0.91 (0.85-0.97)	0.99 (0.93-1.05)	0.94 (0.89-1.00)	0.96 (0.92-1.01)	Replaced	1.15 (1.07-1.23)
Replace MVPA with	0.71 (0.58-1.88)	0.79 (0.73-0.86)	0.86 (0.80-0.93)	0.82 (0.76-0.90)	0.84 (0.78-0.91)	0.87 (0.81-0.94)	Replaced
Partition model^c^	0.84 (0.69-1.03)	0.94 (0.88-0.99)	1.02 (0.97-1.07)	0.97 (0.92-1.03)	0.99 (0.97-1.02)	1.03 (0.99-1.07)	1.18 (1.11-1.26)

Abbreviations: LPA-Home, standing or walking around at home; LPA-Work, standing or walking around at work; MVPA, moderate-to-vigorous physical activity; OR, odds ratio; SB-Home, other sitting at home; SB-Work, sitting at work or away from home or driving.

^a^ Total time is the sum of time spent on sitting watching television, SB-Work, SB-Home, LPA-Home, LPA-Work, and MVPA. ORs are adjusted for age (years); education (registered nurse, bachelor, or graduate); marital status (married, widowed, or separated or divorced); household income (quintiles); family history of cancer, myocardial infarction, and diabetes (yes or no); baseline hypertension and high cholesterol (yes or no); menopausal status and postmenopausal hormone use (premenopausal, postmenopausal and never user, postmenopausal and past user, postmenopausal and current user); aspirin use (regular use or not); smoking history (never, former, current), alcohol intake (none, 1-14.9, ≥15 g/d), total energy intake (quintiles), diet quality (Alternate Healthy Eating Index score, quintiles), sleep duration (≤5, 6, 7, 8, ≥9 hours per day), and body mass index (calculated as weight in kilograms divided by height in meters squared and categorized as <18.5, 18.5-24.9, 25-29.9, ≥30).

^b^ Standardized as normal-pace walking time (hours per day).

^c^ Each OR represents a comparison of healthy aging for every 1 hour per day increase in the exposure variable, not restricting total time nor controlling the displacement of other activity time.

### SB, LPA, and 4 Domains of Healthy Aging

The associations of the 5 exposures with each domain of healthy aging are shown in eTable 4 and eFigure 4 in Supplement 1. Time spent watching television was negatively associated with odds of each domain of healthy aging. In contrast, time spent on LPA-Home and LPA-Work were associated with higher odds of each domain of healthy aging. In addition, the above replacement outcomes also were observed in all 4 domains of healthy aging (eTable 5 in Supplement 1).

### Sensitivity Analysis

In secondary analyses excluding women who died before the end of follow-up, we found similar results for the independent association of all 5 exposures and also observed similar replacement outcomes (eTable 6 in Supplement 1). When performing sensitivity analyses restricted to participants with complete data or using multiple imputations for missing data, no significant changes of replacement outcomes were observed (eTable 7 in Supplement 1).

## Discussion

To our knowledge, this cohort study is the first prospective cohort study to examine the independent and replacement associations of SB and LPA with healthy aging. We found that SB, especially television watching, was significantly associated with lower odds of healthy aging; meanwhile, LPA-Work was associated with higher odds of healthy aging. These associations were consistent for 4 different domains of healthy aging. We further found that replacing television watching with other SBs, LPA-Work, LPA-Home, MVPA, or sleep (in participants who slept ≤7 hours per day) was all associated with increased odds of healthy aging. Interestingly, replacing SB-Work with LPA-Work was also associated with increased odds of healthy aging. These findings suggest that both LPA at home and at work are better than SB, and MVPA may lead to stronger odds of achieving healthy aging.

### Explanations and Potential Mechanism

The independent association of SB with healthy aging is consistent with previous studies of prolonged sitting, particularly watching television, in relation to multiple diseases^38,39,40^ and mortality^41^ and are also consistent with previous analysis in the NHS showing a positive association between SB and chronic diseases.^18,22^ Moreover, television watching was the strongest negative risk factor associated with healthy aging among several SBs, whereas there was no significant association of SB-Work or SB-Home with healthy aging; these findings verified the different associations of different sedentary behaviors with overall health. In addition, we found a protective association of LPA and MVPA with healthy aging, which not only extends the current evidence on the benefits of LPA^12,42,43^ and MVPA^29,44^ but also indicates a continuum in the associations of physical activity levels with overall health.

There are several potential mechanisms for the observed association between SB and healthy aging. First, prolonged sitting may affect physical function by causing distinctive cellular and molecular responses in the skeletal muscle that impairs its function and mitochondrial activity.^45^ Skeletal muscles are known to play an important role in controlling glucose homeostasis. Meanwhile, excess sitting may affect chronic diseases by reducing insulin sensitivity, disrupting postprandial glucose and lipid metabolism,^46^ and increasing inflammation.^47,48^ Second, prolonged sitting may negatively impact the peripheral and central vascular markers,^49^ such as the cerebral blood flow,^50^ which may explain the negative association of television watching and different domains of healthy aging in this study. Third, television watching typically displaces physical activity and thus reduces energy expenditure. Also, studies have reported that individuals who spend more time watching television tend to follow unhealthy eating patterns^51^ and increase total energy intake,^52^ which have direct associations with disease risk. In our study, women who spent more time watching television tended to have less physical activity, but the associations of television watching, LPA, and MVPA with the development of healthy aging were largely independent. The combination of these reasons may explain our findings that television watching was more strongly negatively associated with healthy aging than other SBs.

We found that replacing watching television with either LPA or MVPA might promote healthy aging, and the greater the intensity of physical activity, the stronger the association. These findings are complementary to previous studies that have found replacing SB with LPA and MVPA are associated with reduced mortality.^19,53^ Moreover, we found that replacing SB-Work with LPA-Work also had a beneficial association with healthy aging. These findings indicate that physical activity need not be high intensity to potentially benefit various aspects of health, which has especially important public health implications, as older people tend to have limited physical ability to engage in MVPA. Moreover, our study found that 61% (53%-68%) of usual agers could be attributable to the joint effects of 4 factors: television watching at least 3 hours per day, LPA-Work less than 3 hours per day, less than 30 minutes per day of standardized MVPA, and overweight. These findings provide important evidence that a more meaningful reallocation of time in terms of daily activities could have significant implications for individuals and public health.

However, due to technological advances and lifestyle changes, SB has increased greatly among older adults.^54^ In the US, 84% of older adults spent 2 or more hours per day sitting watching television,^55^ 25.7% reported sitting for more than 8 hours per day, and 44.6% were inactive.^56^ It is more concerning that the prevalence of sitting for more than 8 hours per day and being inactive increased with age.^56^ Given the strong association observed between sedentary lifestyle and healthy aging, public health campaigns to promote health should not only promote increasing physical activities, but also decreasing sedentary behaviors, especially prolonged television watching. Furthermore, physical activity comprises the sum of nonexercise activities (eg, housework and gardening) and exercise activities (eg, running and weight training). Importantly, these nonexercise activities contribute a much larger proportion to overall energy expenditure than planned exercise does on a daily basis.^47^ Our study found that substituting SB, even if only with LPAs, such as standing or walking around at home (which likely reflects household work) or at work, was associated with significantly higher odds of healthy aging.

### Strengths and Limitations

Our study has several strengths. We investigated diverse sedentary behaviors and LPA in association of healthy aging after 20 years’ follow-up with a high follow-up rate, which minimized the likelihood of recall and selection biases. There are several limitations as well. First, measures of different behaviors were all self-reported so were less accurate than objective measurement methods, and not all participants could report time use for all 24 hours per day. However, objective methods cannot distinguish the context of the activity. For example, they cannot distinguish sitting watching television vs sitting at work. However, self-reports can distinguish specific types of SB, LPA at home or at work, and also are better suited to large cohort studies. Our questionnaire to measure physical activity has been validated in a similar population and has shown reasonable accuracy.^23,24,27^ Moreover, since the exposure information was collected before any of the study outcomes occurred, the measurement errors would most likely be nondifferential and bias true associations toward the null. Also, assessment of LPA is more challenging than that of MVPA^57^; thus, greater measurement error for LPA may have attenuated the observed associations with LPA. Single exposure assessment at baseline may not capture the long-term dynamic pattern of these activities. Moreover, the observational nature of this study cannot prove a causal relationship of television watching and LPA with healthy aging. Furthermore, as our study population was confined to US nurses, our findings might not be generalizable to other populations.

## Conclusions

In this large cohort study, we found that SBs, especially prolonged television watching, were associated with decreased odds of healthy aging. In contrast, LPA was associated with significantly increased odds of health aging, and MVPA was associated with even higher odds of achieving healthy aging. Replacing television watching with MVPA or even LPA, or sleep (in participants who slept ≤7 hours per day) was all associated with improved healthy aging. These findings complement previous evidence on the association between these behaviors and mortality, and provide important evidence for promoting active lifestyles for achieving optimal health at older ages.

## Notes

Supplement 1. **eTable 1.** Age-Adjusted Baseline Characteristics of the Included and Excluded Participants **eTable 2.** Odds of Healthy Aging Among Women According to Sedentary Behaviors and Light-Intensity Physical Activities in Hours per Week, Stratified by Age Groups **eTable 3.** Percentage of Usual Agers Who Can be Potentially Prevented by Adopting Active Lifestyles **eTable 4.** Sedentary Behaviors, Light-Intensity Physical Activities, and 4 Domains of Healthy Aging **eTable 5.** Odds of 4 Dimensions of Healthy Aging According to Isotemporal Substitution of 1 Hour/Day of 6 Activities **eTable 6.** Sensitivity Analysis Among Survivors: Odds of Healthy Aging According to Isotemporal Substitution of 1 Hour/Day of 6 Activities **eTable 7.** Sensitivity Analysis for Missing Data: Odds of Healthy Aging According to Isotemporal Substitution of 1 Hour/Day of 6 Activities **eFigure 1.** Flowchart of Participants **eFigure 2.** Design and Analysis Framework **eFigure 3.** Association of TV Time and Light-Intensity Physical Activities With Healthy Aging, Stratified Analysis by MVPA **eFigure 4.** Odds of Heathy Aging and Its 4 Domains Associated With Sedentary Behaviors and Physical Activities Among Women **eAppendix 1.** Assessment of Healthy Aging **eAppendix 2.** Statistical Methods
eReferences.

Supplement 2.
Data Sharing Statement

## References

[1] Dzau VJ, Inouye SK, Rowe JW, Finkelman E, Yamada T. Enabling healthful aging for all—the National Academy of Medicine grand challenge in healthy longevity. N Engl J Med. 2019;381(18):1699-1701. doi:10.1056/NEJMp1912298 31633895

[2] Chang AY, Skirbekk VF, Tyrovolas S, Kassebaum NJ, Dieleman JL. Measuring population ageing: an analysis of the Global Burden of Disease Study 2017. Lancet Public Health. 2019;4(3):e159-e167. doi:10.1016/S2468-2667(19)30019-2 30851869 PMC6472541

[3] Sun Q, Townsend MK, Okereke OI, Franco OH, Hu FB, Grodstein F. Adiposity and weight change in mid-life in relation to healthy survival after age 70 in women: prospective cohort study. BMJ. 2009;339:b3796. doi:10.1136/bmj.b3796 19789407 PMC3230231

[4] Shi H, Huang T, Ma Y, Eliassen AH, Sun Q, Wang M. Sleep duration and snoring at midlife in relation to healthy aging in women 70 years of age or older. Nat Sci Sleep. 2021;13:411-422. doi:10.2147/NSS.S302452 33762862 PMC7982569

[5] Depp CA, Jeste DV. Definitions and predictors of successful aging: a comprehensive review of larger quantitative studies. Am J Geriatr Psychiatry. 2006;14(1):6-20. doi:10.1097/01.JGP.0000192501.03069.bc 16407577

[6] Schietzel S, Chocano-Bedoya PO, Sadlon A, . Prevalence of healthy aging among community dwelling adults age 70 and older from five European countries. BMC Geriatr. 2022;22(1):174. doi:10.1186/s12877-022-02755-8 35236290 PMC8889763

[7] Daskalopoulou C, Stubbs B, Kralj C, Koukounari A, Prince M, Prina AM. Physical activity and healthy ageing: a systematic review and meta-analysis of longitudinal cohort studies. Ageing Res Rev. 2017;38:6-17. doi:10.1016/j.arr.2017.06.003 28648951

[8] Katzmarzyk PT, Powell KE, Jakicic JM, Troiano RP, Piercy K, Tennant B; 2018 PHYSICAL ACTIVITY GUIDELINES ADVISORY COMMITTEE*. Sedentary behavior and health: update from the 2018 Physical Activity Guidelines Advisory Committee. Med Sci Sports Exerc. 2019;51(6):1227-1241. doi:10.1249/MSS.0000000000001935 31095080 PMC6527341

[9] Li S, Lear SA, Rangarajan S, . Association of sitting time with mortality and cardiovascular events in high-income, middle-income, and low-income countries. JAMA Cardiol. 2022;7(8):796-807. doi:10.1001/jamacardio.2022.1581 35704349 PMC9201743

[10] Amagasa S, Machida M, Fukushima N, . Is objectively measured light-intensity physical activity associated with health outcomes after adjustment for moderate-to-vigorous physical activity in adults: a systematic review. Int J Behav Nutr Phys Act. 2018;15(1):65. doi:10.1186/s12966-018-0695-z 29986718 PMC6038338

[11] Chastin SFM, De Craemer M, De Cocker K, . How does light-intensity physical activity associate with adult cardiometabolic health and mortality: systematic review with meta-analysis of experimental and observational studies. Br J Sports Med. 2019;53(6):370-376. doi:10.1136/bjsports-2017-097563 29695511 PMC6579499

[12] van der Ploeg HP, Chey T, Ding D, Chau JY, Stamatakis E, Bauman AE. Standing time and all-cause mortality in a large cohort of Australian adults. Prev Med. 2014;69:187-191. doi:10.1016/j.ypmed.2014.10.004 25456805

[13] Dogra S, Stathokostas L. Sedentary behavior and physical activity are independent predictors of successful aging in middle-aged and older adults. J Aging Res. 2012;2012:190654. doi:10.1155/2012/190654 22997579 PMC3446656

[14] Bauman A, Ainsworth BE, Sallis JF, ; IPS Group. The descriptive epidemiology of sitting. A 20-country comparison using the International Physical Activity Questionnaire (IPAQ). Am J Prev Med. 2011;41(2):228-235. doi:10.1016/j.amepre.2011.05.003 21767731

[15] Du Y, Liu B, Sun Y, Snetselaar LG, Wallace RB, Bao W. Trends in adherence to the physical activity guidelines for americans for aerobic activity and time spent on sedentary behavior among US adults, 2007 to 2016. JAMA Netw Open. 2019;2(7):e197597. doi:10.1001/jamanetworkopen.2019.7597 31348504 PMC6661709

[16] Grgic J, Dumuid D, Bengoechea EG, . Health outcomes associated with reallocations of time between sleep, sedentary behaviour, and physical activity: a systematic scoping review of isotemporal substitution studies. Int J Behav Nutr Phys Act. 2018;15(1):69. doi:10.1186/s12966-018-0691-3 30001713 PMC6043964

[17] Rees-Punia E, Evans EM, Schmidt MD, . Mortality risk reductions for replacing sedentary time with physical activities. Am J Prev Med. 2019;56(5):736-741. doi:10.1016/j.amepre.2018.12.006 30905483 PMC8485344

[18] Keum N, Cao Y, Oh H, . Sedentary behaviors and light-intensity activities in relation to colorectal cancer risk. Int J Cancer. 2016;138(9):2109-2117. doi:10.1002/ijc.29953 26649988 PMC4885736

[19] Stamatakis E, Rogers K, Ding D, . All-cause mortality effects of replacing sedentary time with physical activity and sleeping using an isotemporal substitution model: a prospective study of 201,129 mid-aged and older adults. Int J Behav Nutr Phys Act. 2015;12:121. doi:10.1186/s12966-015-0280-7 26419654 PMC4589071

[20] Stamatakis E, Gale J, Bauman A, Ekelund U, Hamer M, Ding D. Sitting time, physical activity, and risk of mortality in adults. J Am Coll Cardiol. 2019;73(16):2062-2072. doi:10.1016/j.jacc.2019.02.031 31023430

[21] Mekary RA, Lucas M, Pan A, . Isotemporal substitution analysis for physical activity, television watching, and risk of depression. Am J Epidemiol. 2013;178(3):474-483. doi:10.1093/aje/kws590 23785112 PMC3727339

[22] Hu FB, Li TY, Colditz GA, Willett WC, Manson JE. Television watching and other sedentary behaviors in relation to risk of obesity and type 2 diabetes mellitus in women. JAMA. 2003;289(14):1785-1791. doi:10.1001/jama.289.14.1785 12684356

[23] Chasan-Taber S, Rimm EB, Stampfer MJ, . Reproducibility and validity of a self-administered physical activity questionnaire for male health professionals. Epidemiology. 1996;7(1):81-86. doi:10.1097/00001648-199601000-00014 8664406

[24] Wolf AM, Hunter DJ, Colditz GA, . Reproducibility and validity of a self-administered physical activity questionnaire. Int J Epidemiol. 1994;23(5):991-999. doi:10.1093/ije/23.5.991 7860180

[25] Hu FB, Sigal RJ, Rich-Edwards JW, . Walking compared with vigorous physical activity and risk of type 2 diabetes in women: a prospective study. JAMA. 1999;282(15):1433-1439. doi:10.1001/jama.282.15.1433 10535433

[26] Khalili H, Ananthakrishnan AN, Konijeti GG, . Physical activity and risk of inflammatory bowel disease: prospective study from the Nurses’ Health Study cohorts. BMJ. 2013;347:f6633. doi:10.1136/bmj.f6633 24231178 PMC3935281

[27] Al-Shaar L, Pernar CH, Chomistek AK, . Reproducibility, validity, and relative validity of self-report methods for assessing physical activity in epidemiologic studies: findings from the Women’s Lifestyle Validation Study. Am J Epidemiol. 2022;191(4):696-710. doi:10.1093/aje/kwab294 34999754 PMC9430451

[28] Rowe JW, Kahn RL. Successful aging. Gerontologist. 1997;37(4):433-440. doi:10.1093/geront/37.4.433 9279031

[29] Sun Q, Townsend MK, Okereke OI, Franco OH, Hu FB, Grodstein F. Physical activity at midlife in relation to successful survival in women at age 70 years or older. Arch Intern Med. 2010;170(2):194-201. doi:10.1001/archinternmed.2009.503 20101015 PMC3024209

[30] Ma W, Hagan KA, Heianza Y, Sun Q, Rimm EB, Qi L. Adult height, dietary patterns, and healthy aging. Am J Clin Nutr. 2017;106(2):589-596. doi:10.3945/ajcn.116.147256 28592610 PMC5525116

[31] Li S, Hagan K, Grodstein F, VanderWeele TJ. Social integration and healthy aging among U.S. women. Prev Med Rep. 2018;9:144-148. doi:10.1016/j.pmedr.2018.01.013 29527467 PMC5840846

[32] Guralnik JM, Kaplan GA. Predictors of healthy aging: prospective evidence from the Alameda County study. Am J Public Health. 1989;79(6):703-708. doi:10.2105/AJPH.79.6.703 2729467 PMC1349627

[33] Willett WC, Sampson L, Stampfer MJ, . Reproducibility and validity of a semiquantitative food frequency questionnaire. Am J Epidemiol. 1985;122(1):51-65. doi:10.1093/oxfordjournals.aje.a114086 4014201

[34] Yuan C, Spiegelman D, Rimm EB, . Relative validity of nutrient intakes assessed by questionnaire, 24-hour recalls, and diet records as compared with urinary recovery and plasma concentration biomarkers: findings for women. Am J Epidemiol. 2018;187(5):1051-1063. doi:10.1093/aje/kwx328 29036411 PMC5928456

[35] Chiuve SE, Fung TT, Rimm EB, . Alternative dietary indices both strongly predict risk of chronic disease. J Nutr. 2012;142(6):1009-1018. doi:10.3945/jn.111.157222 22513989 PMC3738221

[36] Spiegelman D, Hertzmark E, Wand HC. Point and interval estimates of partial population attributable risks in cohort studies: examples and software. Cancer Causes Control. 2007;18(5):571-579. doi:10.1007/s10552-006-0090-y 17387622

[37] Katzmarzyk PT, Church TS, Craig CL, Bouchard C. Sitting time and mortality from all causes, cardiovascular disease, and cancer. Med Sci Sports Exerc. 2009;41(5):998-1005. doi:10.1249/MSS.0b013e3181930355 19346988

[38] Chan DSM, Abar L, Cariolou M, . World Cancer Research Fund International: Continuous Update Project—systematic literature review and meta-analysis of observational cohort studies on physical activity, sedentary behavior, adiposity, and weight change and breast cancer risk. Cancer Causes Control. 2019;30(11):1183-1200. doi:10.1007/s10552-019-01223-w 31471762

[39] Ikehara S, Iso H, Maruyama K, Ukawa S, Tamakoshi A; Japan Collaborative Cohort Study. Television viewing time, walking time, and risk of type 2 diabetes in Japanese men and women: the Japan Collaborative Cohort Study. Prev Med. 2019;118:220-225. doi:10.1016/j.ypmed.2018.11.006 30408447

[40] Ford ES, Caspersen CJ. Sedentary behaviour and cardiovascular disease: a review of prospective studies. Int J Epidemiol. 2012;41(5):1338-1353. doi:10.1093/ije/dys078 22634869 PMC4582407

[41] Patel AV, Bernstein L, Deka A, . Leisure time spent sitting in relation to total mortality in a prospective cohort of US adults. Am J Epidemiol. 2010;172(4):419-429. doi:10.1093/aje/kwq155 20650954 PMC3590043

[42] Katzmarzyk PT. Standing and mortality in a prospective cohort of Canadian adults. Med Sci Sports Exerc. 2014;46(5):940-946. doi:10.1249/MSS.0000000000000198 24152707

[43] Jain P, Bellettiere J, Glass N, . The relationship of accelerometer-assessed standing time with and without ambulation and mortality: the WHI OPACH study. J Gerontol A Biol Sci Med Sci. 2021;76(1):77-84. doi:10.1093/gerona/glaa227 33225345 PMC7756713

[44] Hamer M, Lavoie KL, Bacon SL. Taking up physical activity in later life and healthy ageing: the English longitudinal study of ageing. Br J Sports Med. 2014;48(3):239-243. doi:10.1136/bjsports-2013-092993 24276781 PMC3913225

[45] Hamilton MT, Hamilton DG, Zderic TW. Role of low energy expenditure and sitting in obesity, metabolic syndrome, type 2 diabetes, and cardiovascular disease. Diabetes. 2007;56(11):2655-2667. doi:10.2337/db07-0882 17827399

[46] Bellettiere J, Healy GN, LaMonte MJ, . Sedentary behavior and prevalent diabetes in 6,166 older women: the Objective Physical Activity and Cardiovascular Health Study. J Gerontol A Biol Sci Med Sci. 2019;74(3):387-395. doi:10.1093/gerona/gly101 29726906 PMC6376101

[47] Levine JA. Sick of sitting. Diabetologia. 2015;58(8):1751-1758. doi:10.1007/s00125-015-3624-6 26003325 PMC4519030

[48] Howard BJ, Balkau B, Thorp AA, . Associations of overall sitting time and TV viewing time with fibrinogen and C reactive protein: the AusDiab study. Br J Sports Med. 2015;49(4):255-258. doi:10.1136/bjsports-2013-093014 24550208

[49] Credeur DP, Miller SM, Jones R, . Impact of prolonged sitting on peripheral and central vascular health. Am J Cardiol. 2019;123(2):260-266. doi:10.1016/j.amjcard.2018.10.014 30409414

[50] Wheeler MJ, Dunstan DW, Smith B, . Morning exercise mitigates the impact of prolonged sitting on cerebral blood flow in older adults. J Appl Physiol (1985). 2019;126(4):1049-1055. doi:10.1152/japplphysiol.00001.2019 30730813 PMC6485691

[51] Santaliestra-Pasías AM, Mouratidou T, Huybrechts I, . Increased sedentary behaviour is associated with unhealthy dietary patterns in European adolescents participating in the HELENA study. Eur J Clin Nutr. 2014;68(3):300-308. doi:10.1038/ejcn.2013.170 24045790

[52] Braude L, Stevenson RJ. Watching television while eating increases energy intake: examining the mechanisms in female participants. Appetite. 2014;76:9-16. doi:10.1016/j.appet.2014.01.005 24462489

[53] Dohrn IM, Kwak L, Oja P, Sjöström M, Hagströmer M. Replacing sedentary time with physical activity: a 15-year follow-up of mortality in a national cohort. Clin Epidemiol. 2018;10:179-186. doi:10.2147/CLEP.S151613 29416378 PMC5790069

[54] Prince SA, Melvin A, Roberts KC, Butler GP, Thompson W. Sedentary behaviour surveillance in Canada: trends, challenges and lessons learned. Int J Behav Nutr Phys Act. 2020;17(1):34. doi:10.1186/s12966-020-00925-8 32151285 PMC7063715

[55] Yang L, Cao C, Kantor ED, . Trends in sedentary behavior among the US population, 2001-2016. JAMA. 2019;321(16):1587-1597. doi:10.1001/jama.2019.3636 31012934 PMC6487546

[56] Ussery EN, Fulton JE, Galuska DA, Katzmarzyk PT, Carlson SA. Joint prevalence of sitting time and leisure-time physical activity among US adults, 2015-2016. JAMA. 2018;320(19):2036-2038. doi:10.1001/jama.2018.17797 30458482 PMC6248166

[57] Füzéki E, Engeroff T, Banzer W. Health benefits of light-intensity physical activity: a systematic review of accelerometer data of the National Health and Nutrition Examination Survey (NHANES). Sports Med. 2017;47(9):1769-1793. doi:10.1007/s40279-017-0724-0 28393328

