# Supplementary material for: Sedentary Behaviors, Light-Intensity Physical Activity, and Healthy Aging
Source: JAMA Netw Open. 2024 Jun 11;7(6):e2416300. doi: 10.1001/jamanetworkopen.2024.16300 (PMC11167497; doi:10.1001/jamanetworkopen.2024.16300)
Supplement: Supplement 1. — eTable 1. Age-Adjusted Baseline Characteristics of the Included and Excluded Participants eTable 2. Odds of Healthy Aging Among Women According to Sedentary Behaviors and Light-Intensity Physical Activities in Hours per Week, Stratified by Age Groups eTable 3. Percentage of Usual Agers Who Can be Potentially Prevented by Adopting Active Lifestyles eTable 4. Sedentary Behaviors, Light-Intensity Physical Activities, and 4 Domains of Healthy Aging eTable 5. Odds of 4 Dimensions of Healthy Aging According to Isotemporal Substitution of 1 Hour/Day of 6 Activities eTable 6. Sensitivity Analysis Among Survivors: Odds of Healthy Aging According to Isotemporal Substitution of 1 Hour/Day of 6 Activities eTable 7. Sensitivity Analysis for Missing Data: Odds of Healthy Aging According to Isotemporal Substitution of 1 Hour/Day of 6 Activities eFigure 1. Flowchart of Participants eFigure 2. Design and Analysis Framework eFigure 3. Association of TV Time and Light-Intensity Physical Activities With Healthy Aging, Stratified Analysis by MVPA eFigure 4. Odds of Heathy Aging and Its 4 Domains Associated With Sedentary Behaviors and Physical Activities Among Women eAppendix 1. Assessment of Healthy Aging eAppendix 2. Statistical Methods eReferences. [file jamanetwopen-e2416300-s001.pdf]

Supplemental Online Content

Shi H, Hu FB, Huang T, et al. Sedentary behaviors, light-intensity physical activity, and healthy aging. *JAMA Netw Open*. 2024;7(6):e2416300. doi:10.1001/jamanetworkopen.2024.16300

eTable 1. Age-Adjusted Baseline Characteristics of the Included and Excluded Participants
eTable 2. Odds of Healthy Aging Among Women According to Sedentary Behaviors and Light-Intensity Physical Activities in Hours per Week, Stratified by Age Groups
eTable 3. Percentage of Usual Agers Who Can be Potentially Prevented by Adopting Active Lifestyles
eTable 4. Sedentary Behaviors, Light-Intensity Physical Activities, and 4 Domains of Healthy Aging
eTable 5. Odds of 4 Dimensions of Healthy Aging According to Isotemporal Substitution of 1 Hour/Day of 6 Activities
eTable 6. Sensitivity Analysis Among Survivors: Odds of Healthy Aging According to Isotemporal Substitution of 1 Hour/Day of 6 Activities
eTable 7. Sensitivity Analysis for Missing Data: Odds of Healthy Aging According to Isotemporal Substitution of 1 Hour/Day of 6 Activities
eFigure 1. Flowchart of Participants
eFigure 2. Design and Analysis Framework
eFigure 3. Association of TV Time and Light-Intensity Physical Activities With Healthy Aging, Stratified Analysis by MVPA
eFigure 4. Odds of Heathy Aging and Its 4 Domains Associated With Sedentary Behaviors and Physical Activities Among Women
eAppendix 1. Assessment of Healthy Aging
eAppendix 2. Statistical Methods
eReferences.

This supplemental material has been provided by the authors to give readers additional information about their work.

© 2024 Shi H et al. *JAMA Network Open.*

eTable 1. Age-adjusted baseline characteristics of the included and excluded participants

Characteristics	Included (*n* = 45 176)	Excluded [b] (*n* = 11 910)
Age, years[a]	59.2 (6.0)	60.7 (5.9)
Education, %		
Registered nurse	70.3	72.4
Bachelor	19.8	19.1
Master or doctorate	9.9	8.5
Husband's education, %		
≤ High school	45.8	46.2
College graduate	29.5	29.1
Graduate school	24.8	24.7
Race(white), %	97.8	96.2
Marital status, %		
Married	81.7	81.0
Widowed	10.7	10.9
Separated, divorced, or never married	7.7	8.2
Family annual income, 10,000$	6.0 (4.7-7.7)	6.0 (4.6-7.8)
BMI, kg/m^2	25.8 (4.8)	26.1 (4.9)
Smoking status, %		
Never smoker	44.1	47.0
Past smoker	41.4	39.5
Current smoker	14.5	13.5
Alcohol intake, %		
None	44.9	47.7
1-14.9 g/d	44.8	43.1
≥15 g/d	10.3	9.2
Diet quality (AHEI score)	48.3 (10.5)	48.1 (10.6)
Total energy intake, kcal	1742.7 (518.3)	1746.6 (527.4)
Moderate-to-vigorous physical activity (MET-h/wk)	12.1 (4.6-25.9)	11.7 (4.2-24.9)
Light-intensity physical activity, h/day	4.9 (2.4-8.4)	4.9 (2.3-8.4)
Sedentary time, h/day	4.5 (2.8-7.1)	4.5 (2.8-6.6)
Sleep duration, h/day	7.0 (1.0)	6.9 (1.0)
Family history of diabetes, %	28.2	28.7
Family history of myocardial infarction, %	18.3	18.0
Family history of cancer, %	13.9	13.3
Hypertension, %	23.0	25.1
High cholesterol, %	33.2	35.5
Use of multivitamin, %	43.3	45.0
Menopausal status and hormone use, %		
Pre-menopausal	8.9	8.6
Post and never used	49.0	51.3
Post and past user	3.2	3.2
Post and current user	38.8	36.9
Regular aspirin, ≥ 2 tabs/week, %	28.8	28.1

Values are means (SD) or medians (P_{25}, P_{75}) for continuous variables, percentages for categorical variables; and are standardized to the age distribution of the study population.

[a] Value is not age adjusted.

[b] Excluded because missing heathy aging status.

© 2024 Shi H et al. *JAMA Network Open.*

eTable 2. Odds ratios of healthy aging among women according to sedentary behaviors and light-intensity physical activities in hours per week, stratified by age groups[a]

Age 1992 (Age 2012)	Categories	OR (95%CI)				
		Sitting watching TV	Sitting at work or away from home or driving	Other sitting at home	Standing or walking around at home	Standing or walking around at work
50- (70-)	0-1h	1.00	1.00	1.00	1.00	1.00
50- (70-)	2-5h	0.99 (0.84-1.19)	0.89 (0.70-1.14)	1.10 (0.84-1.44)	1.14 (0.74-1.76)	1.39 (1.00-1.93)
50- (70-)	6-20h	0.99 (0.85-1.18)	0.89 (0.70-1.12)	1.06 (0.82-1.38)	1.34 (0.88-2.02)	1.42 (1.04-1.95)
50- (70-)	21-40h	0.86 (0.68-1.08)	0.86 (0.66-1.10)	0.94 (0.69-1.28)	1.33 (0.87-2.01)	1.63 (1.19-2.24)
50- (70-)	≥41h	0.86 (0.51-1.46)	0.93 (0.67-1.29)	1.16 (0.71-1.90)	1.36 (0.89-2.10)	1.57 (1.13-2.19)
55- (75-)	0-1h	1.00	1.00	1.00	1.00	1.00
55- (75-)	2-5h	0.94 (0.75-1.19)	1.05 (0.79-1.41)	1.28 (0.87-1.87)	1.82 (0.92-3.57)	0.99 (0.69-1.41)
55- (75-)	6-20h	0.79 (0.63-0.99)	1.02 (0.77-1.36)	1.20 (0.83-1.75)	1.88 (0.97-3.63)	1.07 (0.76-1.49)
55- (75-)	21-40h	0.50 (0.36-0.69)	0.97 (0.71-1.34)	1.07 (0.70-1.63)	1.93 (0.99-3.77)	1.21 (0.86-1.69)
55- (75-)	≥41h	0.56 (0.29-1.09)	0.79 (0.49-1.29)	0.96 (0.53-1.76)	1.91 (0.97-3.76)	1.28 (0.89-1.84)
60- (80-)	0-1h	1.00	1.00	1.00	1.00	1.00
60- (80-)	2-5h	0.77 (0.54-1.09)	1.29 (0.90-1.85)	1.81 (0.96-3.41)	1.41 (0.59-3.36)	0.87 (0.60-1.27)
60- (80-)	6-20h	0.63 (0.45-0.88)	0.98 (0.68-1.41)	1.28 (0.69-2.39)	1.23 (0.53-2.85)	0.95 (0.67-1.35)
60- (80-)	21-40h	0.52 (0.34-0.78)	1.05 (0.66-1.65)	1.35 (0.70-2.60)	1.26 (0.54-2.95)	1.19 (0.82-1.73)
60- (80-)	≥41h	0.28 (0.10-0.78)	0.97 (0.46-2.01)	1.28 (0.55-2.98)	1.46 (0.62-3.45)	1.32 (0.85-2.05)
$P_{interaction}$		0.001	0.50	0.47	0.83	0.35

[a] Adjusted for age (years), education (registered nurse, bachelor, or graduate), marital status (married, widowed, or separated/divorced), household income (quintiles); family history of cancer, myocardial infarction and diabetes (yes or no); baseline hypertension and high cholesterol (yes or no); menopausal status and postmenopausal hormone use (pre-menopausal, post-menopausal and never user, post-menopausal and past user, post-menopausal and current user), aspirin use (regular use or not); smoking history (never, former smoker, current smoker), alcohol intake (none, 1-14.9, ≥15 g/d), total energy intake (quintiles), diet quality (Alternate Healthy Eating Index score, quintiles), sleep duration (≤5, 6, 7, 8, ≥9h), moderate or vigorous-intensity activity (MET-hours/week, in quintiles), body mass index (<18.5, 18.5-24.9, 25-29.9, ≥30 kg/m^2).

© 2024 Shi H et al. *JAMA Network Open.*

eTable 3. Percentage of usual agers that can be potentially prevented by adopting active lifestyles

Lifestyle factors	PAR (95%CI) [a]
X1: Sitting watching TV < 3 hour/day	0.063 (0.035-0.091)
X2: Standing or walking around at work ≥3 hours/day	0.121 (0.075-0.166)
X3: Standardized MVPA ≥30 min/day	0.198 (0.163-0.232)
X4: not overweight (BMI<25 kg/m^2)	0.415 (0.380-0.450)
X1+X2+X3+X4	0.607 (0.528-0.676)

[a] PAR: population-attributable risk; MVPA: moderate or vigorous-intensity physical activity; TV: sitting while watching television. Fixed factors: age (years), education (registered nurse, bachelor, or graduate), marital status (married, widowed, or separated/divorced), household income (quintiles); family history of cancer, myocardial infarction and diabetes (yes or no); baseline hypertension and high cholesterol (yes or no); menopausal status and postmenopausal hormone use (pre-menopausal, post-menopausal and never user, post-menopausal and past user, post-menopausal and current user), aspirin use (regular use or not); smoking history (never, former smoker, current smoker), alcohol intake (none, 1-14.9, ≥15 g/d), total energy intake (quintiles), diet quality (Alternate Healthy Eating Index score, quintiles), sleep duration (≤5, 6, 7, 8, ≥9h).

© 2024 Shi H et al. *JAMA Network Open.*

eTable 4. Sedentary behaviors, light-intensity physical activities and 4 domains of healthy aging, Nurses' Health Study (1992-2012)

Time (hours/wk)	OR[a](95%CI), reference: 0-1 hour/wk			
	No main chronic diseases	Good physical function	Good mental health	No impairment of memory
Sitting watching TV				
2-5	0.90 (0.82-0.98)	0.95 (0.85-1.06)	0.98 (0.90-1.08)	1.00 (0.92-1.09)
6-20	0.91 (0.84-0.99)	0.88 (0.79-0.97)	0.92 (0.85-1.00)	1.01 (0.93-1.09)
21-40	0.92 (0.84-1.01)	0.68 (0.59-0.77)	0.78 (0.70-0.86)	0.92 (0.83-1.01)
≥41	0.81 (0.68-0.95)	0.57 (0.43-0.76)	0.56 (0.47-0.67)	0.68 (0.58-0.80)
Sitting at work or away from home or driving				
2-5	1.05 (0.97-1.14)	1.03 (0.90-1.17)	1.13 (1.04-1.23)	1.07 (0.99-1.16)
6-20	1.04 (0.96-1.12)	1.01 (0.89-1.14)	1.19 (1.10-1.29)	1.08 (0.99-1.16)
21-40	1.05 (0.96-1.16)	1.01 (0.88-1.16)	1.23 (1.11-1.35)	1.07 (0.97-1.17)
≥41	1.09 (0.95-1.25)	0.93 (0.76-1.13)	1.26 (1.09-1.45) [b]	0.99 (0.87-1.15)
Other sitting at home				
2-5	0.98 (0.88-1.10)	1.06 (0.90-1.24)	1.15 (1.02-1.29)	1.03 (0.92-1.15)
6-20	0.94 (0.84-1.05)	1.01 (0.86-1.18)	1.12 (0.99-1.25)	1.02 (0.92-1.14)
21-40	0.95 (0.84-1.07)	0.89 (0.75-1.07)	1.02 (0.90-1.15)	1.01 (0.89-1.14)
≥41	0.95 (0.80-1.12)	0.91 (0.70-1.18)	0.92 (0.77-1.10)	0.79 (0.67-0.93)
Standing or walking around at home				
2-5	1.02 (0.87-1.20)	0.99 (0.78-1.25)	1.09 (0.92-1.28)	1.16 (0.99-1.36)
6-20	1.03 (0.88-1.21)	1.03 (0.82-1.28)	1.15 (0.98-1.34)	1.22 (1.05-1.42)
21-40	1.10 (0.94-1.29)	1.01 (0.80-1.27)	1.23 (1.04-1.44)	1.29 (1.10-1.50)
≥41	1.20 (1.02-1.41)	1.02 (0.81-1.29)	1.26 (1.06-1.48)	1.28 (1.09-1.50)
Standing or walking around at work				
2-5	1.10 (1.00-1.20)	1.13 (0.97-1.31)	1.17 (1.07-1.28)	1.19 (1.10-1.30)
6-20	1.16 (1.06-1.26)	1.19 (1.03-1.37)	1.34 (1.23-1.47)	1.28 (1.18-1.39)
21-40	1.19 (1.09-1.30)	1.36 (1.18-1.58)	1.57 (1.43-1.72)	1.36 (1.25-1.48)
≥41	1.17 (1.06-1.29)	1.39 (1.19-1.63)	1.49 (1.34-1.66)	1.33 (1.21-1.47)

[a] Adjusted for age (years), education (registered nurse, bachelor, or graduate), marital status (married, widowed, or separated/divorced), household income (quintiles); family history of cancer, myocardial infarction and diabetes (yes or no); baseline hypertension and high cholesterol (yes or no); menopausal status and postmenopausal hormone use (pre-menopausal, post-menopausal and never user, post-menopausal and past user, post-menopausal and current user), aspirin use (regular use or not); smoking history (never, former smoker, current smoker), alcohol intake (none, 1-14.9, ≥15 g/d), total energy intake (quintiles), diet quality (Alternate Healthy Eating Index score, quintiles), sleep duration (≤5, 6, 7, 8, ≥9h), moderate or vigorous-intensity physical activity (MET-hours/week, in quintiles); body mass index (BMI <18.5, 18.5-24.9, 25-29.9, ≥30 kg/m^2).

[b] Sitting time at work has different effects on different dimensions of healthy aging.

© 2024 Shi H et al. *JAMA Network Open.*

eTable 5. Odds ratios[a] of 4 dimensions of healthy aging according to isotemporal substitution of 1 hour/day of 6 activities[b], Nurses' Health Study (1992–2012), *n*=45 176

Dimension	TV watching	Sitting at work or away from home or driving	Other sitting at home	Standing or walking around at home	Standing or walking around at work	MVPA [c]
Y1	Substitution model					
	Replaced	1.01 (0.99-1.03)	1.00 (0.98-1.03)	1.03 (1.01-1.05)	1.02 (1.00-1.03)	1.05 (1.03-1.08)
	0.99 (0.97-1.01)	Replaced	0.99 (0.97-1.01)	1.02 (1.00-1.03)	1.00 (0.99-1.02)	1.03 (1.01-1.06)
	1.00 (0.97-1.02)	1.01 (0.99-1.03)	Replaced	1.02 (1.01-1.04)	1.01 (0.99-1.03)	1.04 (1.02-1.07)
	0.97 (0.95-0.99)	0.98 (0.97-0.99)	0.98 (0.96-0.99)	Replaced	0.99 (0.97-0.99)	1.02 (0.99-1.04)
	0.98 (0.97-1.00)	1.00 (0.98-1.01)	0.99 (0.97-1.01)	1.01 (1.00-1.03)	Replaced	1.03 (1.01-1.06)
	0.95 (0.93-0.98)	0.97 (0.94-0.99)	0.96 (0.94-0.98)	0.98 (0.96-1.00)	0.97 (0.95-0.99)	Replaced
	Partition model					
	0.99 (0.98-1.00)	1.00 (0.99-1.02)	1.00 (0.98-1.01)	1.02 (1.01-1.03)	1.01 (0.99-1.02)	1.04 (1.02-1.06)
Y2	Substitution model					
	Replaced	1.08 (1.05-1.11)	1.08 (1.04-1.12)	1.08 (1.06-1.11)	1.11 (1.08-1.14)	1.35 (1.30-1.39)
	0.93 (0.90-0.95)	Replaced	1.00 (0.97-1.03)	1.00 (0.98-1.02)	1.03 (1.01-1.05)	1.25 (1.21-1.29)
	0.92 (0.89-0.96)	0.99 (0.97-1.03)	Replaced	1.00 (0.98-1.03)	1.03 (1.00-1.05)	1.25 (1.21-1.29)
	0.92 (0.90-0.95)	0.99 (0.98-1.02)	0.99 (0.97-1.02)	Replaced	1.03 (1.01-1.05)	1.25 (1.21-1.28)
	0.90 (0.88-0.92)	0.97 (0.95-0.99)	0.97 (0.95-0.99)	0.97 (0.96-0.99)	Replaced	1.21 (1.18-1.25)
	0.74 (0.72-0.77)	0.80 (0.78-0.83)	0.80 (0.78-0.83)	0.80 (0.78-0.83)	0.82 (0.80-0.85)	Replaced
	Partition model					
	0.93 (0.91-0.95)	1.00 (0.98-1.02)	1.00 (0.98-1.02)	1.00 (0.99-1.01)	1.03 (1.02-1.04)	1.25 (1.22-1.28)
Y3	Substitution model					
	Replaced	1.10 (1.08-1.12)	1.06 (1.04-1.08)	1.09 (1.07-1.11)	1.10 (1.09-1.12)	1.21 (1.18-1.24)
	0.91 (0.89-0.93)	Replaced	0.96 (0.94-0.98)	0.99 (0.97-1.00)	1.00 (0.99-1.02)	1.10 (1.07-1.13)
	0.94 (0.92-0.97)	1.04 (1.02-1.06)	Replaced	1.03 (1.01-1.04)	1.04 (1.02-1.06)	1.14 (1.11-1.17)
	0.92 (0.90-0.93)	1.01 (0.99-1.03)	0.97 (0.96-0.99)	Replaced	1.01 (1.00-1.03)	1.11 (1.09-1.14)
	0.91 (0.89-0.92)	0.99 (0.98-1.01)	0.96 (0.95-0.98)	0.99 (0.97-0.99)	Replaced	1.10 (1.07-1.12)
	0.83 (0.80-0.85)	0.91 (0.89-0.93)	0.88 (0.85-0.90)	0.90 (0.88-0.92)	0.91 (0.89-0.93)	Replaced
	Partition model					
	0.94 (0.92-0.95)	1.03 (1.02-1.04)	0.99 (0.98-1.01)	1.02 (1.01-1.03)	1.03 (1.02-1.04)	1.13 (1.11-1.16)
Y4	Substitution model					
	Replaced	1.04 (1.02-1.06)	1.02 (1.00-1.05)	1.05 (1.03-1.06)	1.05 (1.03-1.06)	1.12 (1.10-1.15)
	0.97 (0.95-0.98)	Replaced	0.99 (0.97-1.01)	1.01 (0.99-1.03)	1.01 (0.99-1.03)	1.09 (1.06-1.11)
	0.98 (0.96-0.99)	1.01 (0.99-1.03)	Replaced	1.02 (1.01-1.04)	1.02 (1.01-1.04)	1.10 (1.07-1.13)
	0.96 (0.94-0.97)	0.99 (0.98-1.01)	0.98 (0.96-0.99)	Replaced	1.00 (0.99-1.01)	1.08 (1.05-1.10)
	0.96 (0.94-0.97)	0.99 (0.97-1.01)	0.98 (0.96-0.99)	1.00 (0.99-1.01)	Replaced	1.07 (1.05-1.10)
	0.89 (0.87-0.91)	0.92 (0.90-0.94)	0.91 (0.89-0.93)	0.93 (0.91-0.95)	0.93 (0.91-0.95)	Replaced
	Partition model					
	0.97 (0.96-0.98)	1.00 (0.99-1.02)	0.99 (0.98-1.00)	1.01 (1.01-1.02)	1.01 (1.01-1.02)	1.09 (1.07-1.11)

Y1: No main chronic diseases; Y2: Good physical function; Y3: Good mental health; Y4: No impairment of memory function.

[a] Adjusted for age (years), education (registered nurse, bachelor, or graduate), marital status (married, widowed, or separated/divorced), household income (quintiles); family history of cancer, myocardial infarction and diabetes (yes or no); baseline hypertension and high cholesterol (yes or no); menopausal status and postmenopausal hormone use (pre-menopausal, post-menopausal and never user, post-menopausal and past user, post-menopausal and current user), aspirin use (regular use or not); smoking history (never, former smoker, current smoker), alcohol intake (none, 1-14.9, ≥15 g/d), total energy intake (quintiles), diet quality (Alternate Healthy Eating Index score, quintiles), sleep duration (≤5, 6, 7, 8, ≥9h), and body mass index (BMI <18.5, 18.5-24.9, 25-29.9, ≥30 kg/m^2).

[b] Total time=Sum of time spent on sitting watching TV, sitting at work, other sitting at home, standing or walking around at home, standing or walking around at work, and moderate-to-vigorous physical activity.

[c] Standardized normal-pace walking time (h/day).

© 2024 Shi H et al. *JAMA Network Open.*

eTable 6. Sensitivity analysis among survivors: Odds ratios of healthy aging according to isotemporal substitution of 1 hour/day of 6 activities[a], Nurses' Health Study (1992–2012) [c]

TV watching	Sitting at work or away from home or driving	Other sitting at home	Standing or walking around at home	Standing or walking around at work	MVPA [b]
Substitution model					
Replaced	1.06 (1.02-1.10)	1.06 (1.02-1.11)	1.08 (1.05-1.12)	1.10 (1.06-1.13)	1.29 (1.23-1.34)
0.94 (0.91-0.98)	Replaced	1.00 (0.97-1.04)	1.02 (0.99-1.05)	1.04 (1.01-1.06)	1.21 (1.17-1.26)
0.94 (0.90-0.98)	0.99 (0.96-1.03)	Replaced	1.02 (0.98-1.05)	1.03 (1.00-1.06)	1.21 (1.16-1.26)
0.93 (0.90-0.96)	0.98 (0.96-1.01)	0.98 (0.95-1.02)	Replaced	1.02 (0.99-1.04)	1.19 (1.15-1.23)
0.91 (0.88-0.94)	0.96 (0.94-0.99)	0.97 (0.94-0.99)	0.98 (0.96-1.01)	Replaced	1.17 (1.13-1.21)
0.78 (0.75-0.81)	0.82 (0.79-0.85)	0.83 (0.79-0.86)	0.84 (0.81-0.87)	0.85 (0.82-0.88)	Replaced
Partition model					
0.94 (0.91-0.96)	0.99 (0.97-1.01)	0.99 (0.97-1.02)	1.01 (0.99-1.03)	1.03 (1.01-1.04)	1.20 (1.17-1.24)

Participants who died before the end of follow-up were excluded from usual agers.
[a] Total time=Sum of time spent on sitting watching TV, sitting at work, other sitting at home, standing or walking around at home, standing or walking around at work, and moderate-to-vigorous physical activity.
[b] Standardized normal-pace walking time (h/day).
[c] Adjusted for age (years), education (registered nurse, bachelor, or graduate), marital status (married, widowed, or separated/divorced), household income (quintiles); family history of cancer, myocardial infarction and diabetes (yes or no); baseline hypertension and high cholesterol (yes or no); menopausal status and postmenopausal hormone use (pre-menopausal, post-menopausal and never user, post-menopausal and past user, post-menopausal and current user), aspirin use (regular use or not); smoking history (never, former smoker, current smoker), alcohol intake (none, 1-14.9, ≥15 g/d), total energy intake (quintiles), diet quality (Alternate Healthy Eating Index score, quintiles), sleep duration (≤5, 6, 7, 8, ≥9h), and body mass index (BMI <18.5, 18.5-24.9, 25-29.9, ≥30 kg/m^2).

© 2024 Shi H et al. *JAMA Network Open.*

eTable 7. Sensitivity analysis for missing data: Odds ratios of healthy aging according to isotemporal substitution of 1 hour/day of 6 activities[a], Nurses' Health Study (1992–2012) [c]

TV watching	Sitting at work or away from home or driving	Other sitting at home	Standing or walking around at home	Standing or Walking around at work	MVPA [b]
Among complete cases, *n*=42 368					
Substitution model					
Replaced	1.06 (1.02-1.10)	1.06 (1.01-1.11)	1.08 (1.04-1.11)	1.10 (1.06-1.13)	1.28 (1.22-1.33)
0.95 (0.91-0.98)	Replaced	1.00 (0.97-1.04)	1.02 (0.99-1.05)	1.04 (1.01-1.07)	1.21 (1.16-1.25)
0.94 (0.90-0.99)	0.99 (0.96-1.03)	Replaced	1.01 (0.98-1.05)	1.03 (1.00-1.07)	1.20 (1.15-1.25)
0.93 (0.90-0.96)	0.98 (0.96-1.01)	0.99 (0.95-1.02)	Replaced	1.02 (0.99-1.04)	1.19 (1.14-1.23)
0.91 (0.88-0.94)	0.96 (0.94-0.99)	0.97 (0.94-0.99)	0.98 (0.96-1.00)	Replaced	1.16 (1.12-1.20)
0.78 (0.75-0.82)	0.83 (0.80-0.86)	0.83 (0.80-0.87)	0.84 (0.81-0.87)	0.86 (0.83-0.89)	Replaced
Partition model					
0.94 (0.91-0.97)	0.99 (0.97-1.01)	0.99 (0.97-1.02)	1.01 (0.99-1.02)	1.03 (1.02-1.05)	1.20 (1.16-1.24)
Multiple Imputation, *n*=45 176					
Substitution model					
Replaced	1.06 (1.02-1.10)	1.06 (1.01-1.11)	1.08 (1.04-1.12)	1.10 (1.06-1.13)	1.27 (1.22-1.33)
0.94 (0.90-0.98)	Replaced	1.00 (0.96-1.04)	1.02 (0.99-1.05)	1.04 (1.01-1.06)	1.21 (1.16-1.25)
0.94 (0.90-0.98)	0.99 (0.96-1.04)	Replaced	1.02 (0.98-1.05)	1.03 (1.00-1.07)	1.20 (1.15-1.25)
0.92 (0.89-0.95)	0.98 (0.96-1.01)	0.98 (0.95-1.02)	Replaced	1.02 (0.99-1.04)	1.18 (1.14-1.23)
0.91 (0.88-0.94)	0.97 (0.94-0.99)	0.97 (0.94-0.99)	0.98 (0.96-1.01)	Replaced	1.16 (1.13-1.20)
0.79 (0.76-0.83)	0.84 (0.81-0.88)	0.85 (0.81-0.88)	0.86 (0.83-0.89)	0.88 (0.85-0.91)	Replaced
Partition model					
0.92 (0.90-0.96)	1.02 (0.99-1.05)	0.99 (0.95-1.02)	1.01 (0.99-1.03)	1.04 (1.02-1.06)	1.27 (1.22-1.32)

[a] Total time=Sum of time spent on sitting watching TV, sitting at work, other sitting time at home, standing or walking around at home, standing or walking around at work, and moderate-to-vigorous physical activity.
[b] Standardized normal-pace walking time (h/day).
[c] Adjusted for age (years), education (registered nurse, bachelor, or graduate), marital status (married, widowed, or separated/divorced), household income (quintiles); family history of cancer, myocardial infarction and diabetes (yes or no); baseline hypertension and high cholesterol (yes or no); menopausal status and postmenopausal hormone use (pre-menopausal, post-menopausal and never user, post-menopausal and past user, post-menopausal and current user), aspirin use (regular use or not); smoking history (never, former smoker, current smoker), alcohol intake (none, 1-14.9, ≥15 g/d), total energy intake (quintiles), diet quality (Alternate Healthy Eating Index score, quintiles), sleep duration (≤5, 6, 7, 8, ≥9h), and body mass index (BMI <18.5, 18.5-24.9, 25-29.9, ≥30 kg/m^2).

© 2024 Shi H et al. *JAMA Network Open.*

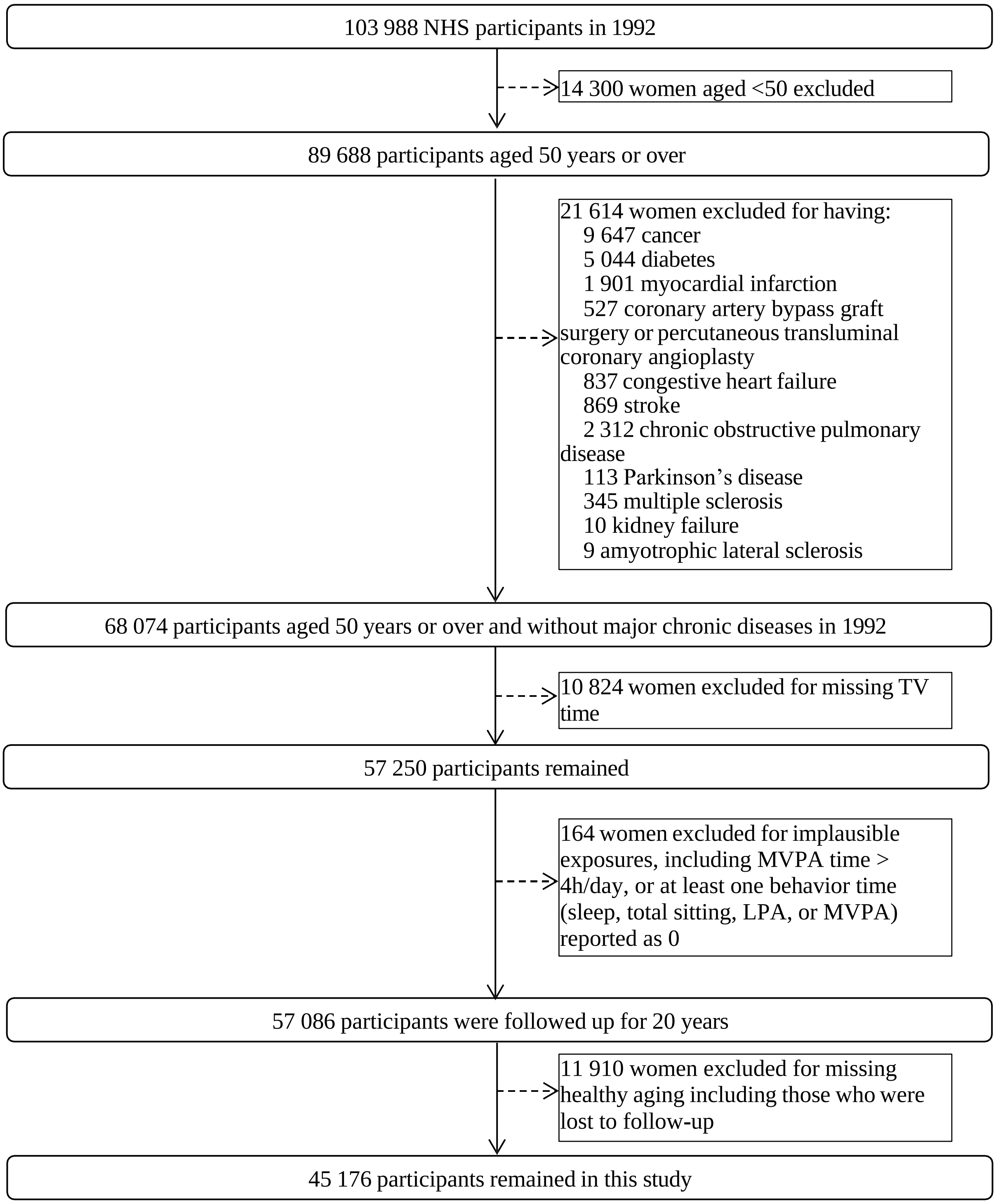

eFigure 1 | Flowchart of participants

© 2024 Shi H et al. *JAMA Network Open.*

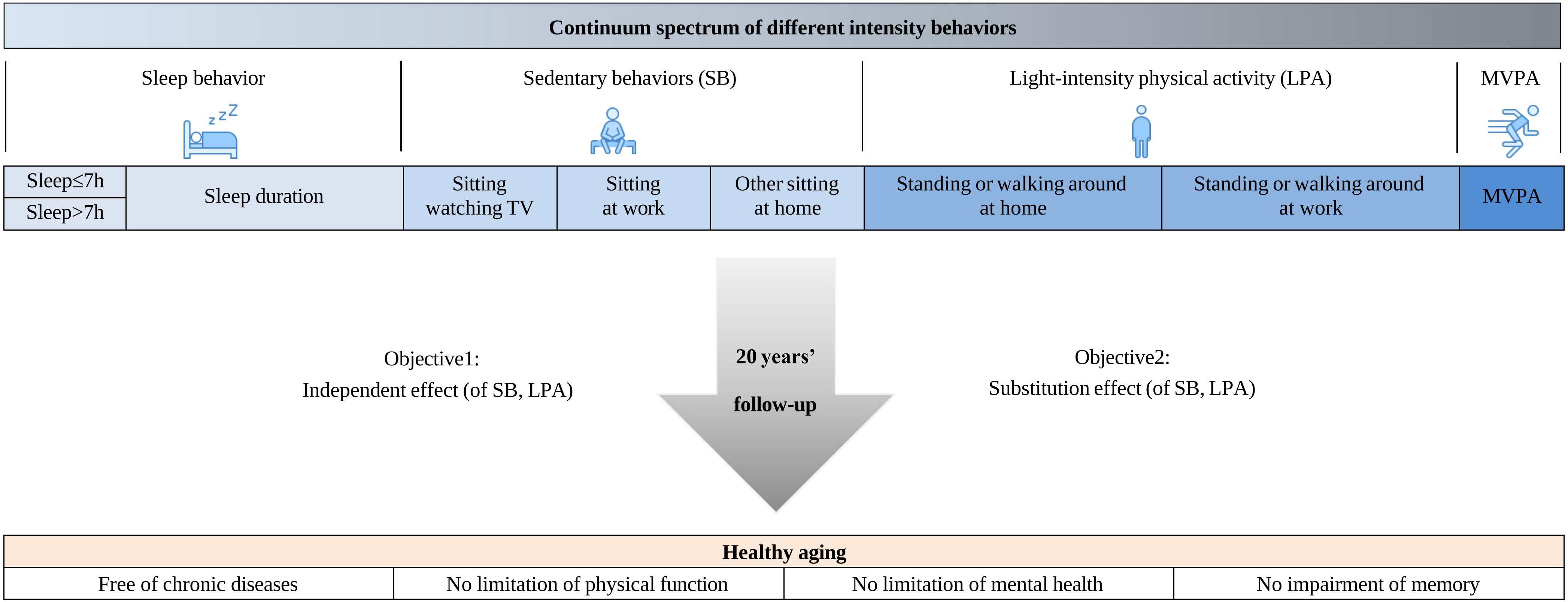

eFigure 2 | Design and analysis framework of this study

© 2024 Shi H et al. *JAMA Network Open.*

eFigure 3 | Association between TV time, light-intensity physical activities and healthy aging, stratified analysis by MVPA[a]

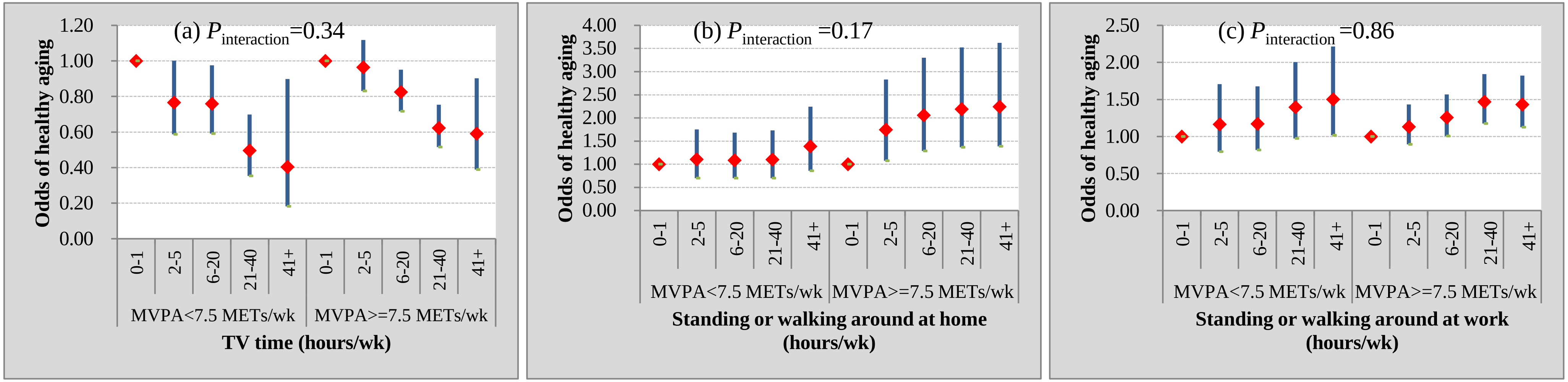

[a] MVPA: moderate or vigorous-intensity physical activity; TV: sitting while watching television. Adjusted for age (years), education (registered nurse, bachelor, or graduate), marital status (married, widowed, or separated/divorced), household income (quintiles); family history of cancer, myocardial infarction and diabetes (yes or no); baseline hypertension and high cholesterol (yes or no); menopausal status and postmenopausal hormone use (pre-menopausal, post-menopausal and never user, post-menopausal and past user, post-menopausal and current user), aspirin use (regular use or not); smoking history (never, former smoker, current smoker), alcohol intake (none, 1-14.9, ≥15g/d), total energy intake (quintiles), diet quality (Alternate Healthy Eating Index score, quintiles), sleep duration (≤5, 6, 7, 8, ≥9h); and body mass index (BMI <18.5, 18.5-24.9, 25-29.9, ≥30 kg/m^2). Physically active (≥7.5) and inactive (<7.5) was defined by using a threshold of 7.5 MET-hours/week, which corresponds to the minimum current physical activity recommendations.

© 2024 Shi H et al. *JAMA Network Open.*

eFigure 4 | Odds ratio of heathy aging and its 4 domains associated with sedentary behaviors and physical activities[a]

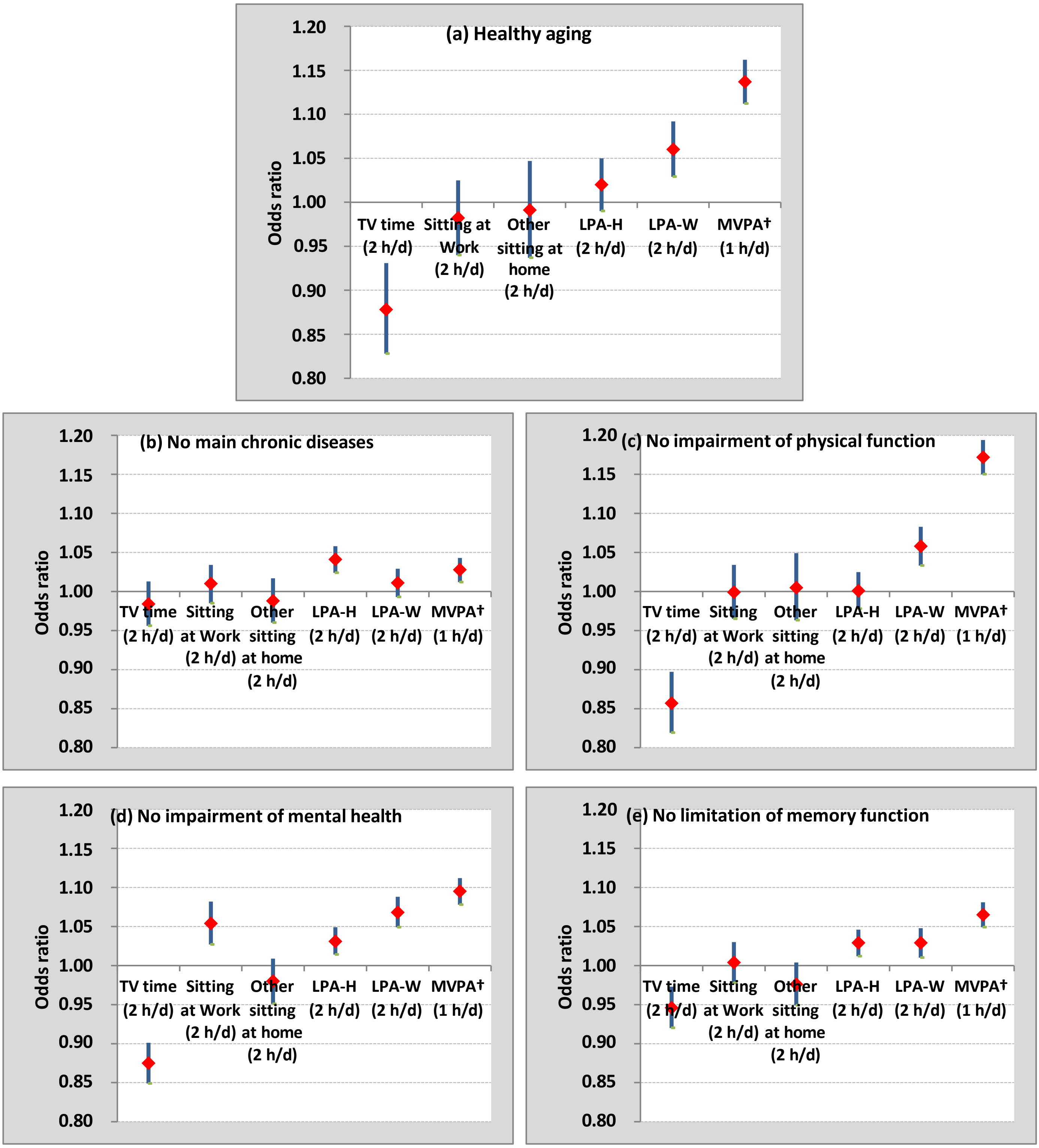

[a] All sedentary behavior variables, LPA and MVPA are included simultaneously in the model. Other sitting at home includes reading, mealtime, and at desk. LPA-H: standing or walking around at home; LPA-W: standing or walking around at work. Error bars indicate 95% confidence intervals. Adjusted for age (years), education (registered nurse, bachelor, or graduate), marital status (married, widowed, or separated/divorced), household income (quintiles); family history of cancer, myocardial infarction and diabetes (yes or no); baseline hypertension and high cholesterol (yes or no); menopausal status and postmenopausal hormone use (pre-menopausal, post-menopausal and never user, post-menopausal and past user, post-menopausal and current user), aspirin use (regular use or not); smoking history (never, former smoker, current smoker), alcohol intake (none, 1-14.9, ≥15 g/d), total energy intake (quintiles), diet quality (Alternate Healthy Eating Index score, quintiles), sleep duration (≤5, 6, 7, 8, ≥9h), body mass index (BMI <18.5, 18.5-24.9, 25-29.9, ≥30 kg/m^2).

[b]MVPA (hours per day) = total MET-hours per week /3 MET (for 1 hour of normal-pace walking) /5 days.

© 2024 Shi H et al. *JAMA Network Open.*

eAppendix 1. Assessment of healthy aging

In order to comprehensively assess the health status of the respondents, based on the concept of successful aging proposed by Rowe and Kahn[1] and other related studies[2-5], we defined healthy aging as surviving to at least 70 years old with maintenance of 4 health domains including being free of 11 main chronic diseases, no impairment of physical, memory and mental health. Participants who did not meet these 4 domains or died during the 20 years' follow-up were classified as usual agers [6].

Information of main chronic diseases was reported on the biennial questionnaires (including cancer, diabetes, myocardial infarction, coronary artery bypass graft surgery or percutaneous transluminal coronary angioplasty, congestive heart failure, stroke, kidney failure, chronic obstructive pulmonary disease, Parkinson's disease, multiple sclerosis, and amyotrophic lateral sclerosis). Study physicians blinded to the participants' exposure status confirmed self-reported diagnosis through medical record review, pathology report review, telephone interview, or supplementary questionnaire inquiries, and self-reported diagnoses were shown to have high validity in this cohort [2]. Women who did not report these 11 diseases at the end of follow-up were considered as being free of chronic diseases.

Physical function was assessed by 10 questions in the Medical Outcomes Study Short-Form Health Survey (SF-36). We inquired about physical limitations in performing different kinds of activities. Each activity had three response choices: "Yes, limited a lot," "Yes, limited a little," or "No, not limited at all." Impairment of physical function was defined as limited at least "a little" on ≥1 moderate activities (such as moving a table, bowling, or pushing a vacuum cleaner; climbing one flight of stairs; walking more than a mile; walking several blocks; bathing or dressing); or limited "a lot" on ≥1 more difficult activities (vigorous activities, like running, lifting heavy objects; lifting or carrying groceries; climbing several flights of stairs; bending, kneeling, or stooping); otherwise, participants were considered as having no impairment of physical function[3]. The validity and reliability of the SF-36 and its components have been previously established[7].

Mental status was evaluated by using the Geriatric Depression Scale-15 (GDS-15). The score on this scale ranges from 1 to 15, with lower scores representing better mental health [8]. No mental health limitation was defined as a GDS-15 score less than or equal to 1 (the median value in this cohort) [3,4].

Lastly, based on the Structured Telephone Interview for Dementia Assessment [9], memory function was assessed through seven questions including general memory; remembering short lists; remembering things from one second to the next; remembering recent events; understanding or following spoken instructions; following a group conversation or a plot in a TV program; and navigating familiar streets. These items were strongly associated with objective cognitive function [10], and have been used to identify individuals with possible cognitive impairment [9]. No impairment in memory was defined as no more than one memory complaint [3,4].

© 2024 Shi H et al. *JAMA Network Open.*

eAppendix 2. Statistical methods

Because the outcome of this study was whether healthy aging is achieved, and the assessment of all four dimensions was only available after 20 years of follow-up, and the specific occurrence time of the outcome cannot be obtained, we used logistic regression models (instead of Cox regression models) to evaluate the independent association between various behaviors and healthy aging. To be consistent with previous studies [11], all exposures were categorized into five groups: 0-1, 2-5, 6-20, 21-40, and ≥41 hours/week. Tests for linear trend were conducted by assigning the median score for each category and treating the median score as a continuous variable. Secondly, to compare the independent role of all five exposures and MVPA as in a prior study [11], we fit a mutually adjusted model (with these behaviors included simultaneously); in this model, all exposures were treated as continuous variables (per 2 hours/day). Because the major type of MVPA was walking and the most common walking-pace was within 2-2.9mph in this cohort [12], we standardized the total MVPA as walking time at this pace by using the formula: total MVPA(MET-hours per week)/3(MET per one hour of walking at 2-2.9mph pace) /5(days per week).

In the above analyses, we adjusted for age, education, marital status, annual household income (estimated from the census tract of participant's residence, geocoded to the 1994 U.S. Census); family history of cancer, myocardial infarction and diabetes; baseline hypertension and high cholesterol; menopausal status and postmenopausal hormone use, aspirin use; smoking history, alcohol intake, total energy intake and diet quality assessed by a validated semi-quantitative food frequency questionnaire[13-15], and sleep duration. Considering that BMI could be in the causal pathway between 24-hour behaviors and healthy aging, and to explore the degree to which the exposure-outcome relationships are explained by BMI, we ran an additional model further adjusting for BMI. Considering the important influence of age on healthy ager's evaluation (especially surviving to the end of follow-up), we examined the association between TV time and healthy aging stratified by age (50-, 55-, ≥60 years at baseline, corresponding to 70-, 75-, ≥80 years at follow-up). Moreover, we calculated the population-attributable risk (PAR), an estimate of the percentage of healthy agers during follow-up that could have been achieved if they engaged in the low risk category for these exposures, assuming that the observed associations were causal[16].

We fitted the ISM to quantify the associations of replacing one hour of one behavior for equal amount of another behavior on healthy aging while the total amount of time of all behaviors was kept constant. Due to the ISM requirement for a linear association between each exposure and the outcome, we modeled sleep as a piecewise variable with a cutoff at 7 h (≤7 h/day and >7 h/day)[17,18] to account for the potential nonlinear, U-shaped association between sleep duration and healthy aging[5]. The Isotemporal Substitution Model is originally expressed as a basic simple model as follows:

Healthy aging risk = (b0) sitting watching TV + (b1) sitting at work + (b2) other sitting at home +(b3) LPA at work +(b4) LPA at home + (b5) MVPA + (b6) sleep +(b7) total time* + (b8) covariates

By eliminating one behavior from the model (for example, sitting watching TV), the coefficient (b3) represents the effect of substituting 60 minutes/day of LPA at work for 60 minutes/day of sitting watching TV, while holding total time constant; the coefficient (b4) represents the effect of substituting 60 minutes/day of LPA at home for 60 minutes/day of TV-watching while holding total time constant; and so on. The coefficient (b7) for total time represents the omitted behavior (herein, time spent on sitting watching TV). Similar interpretation for the remaining substitution models can be applied when other behaviors are omitted from the model.

For comparison, we also fitted the partition model, which partitions "total time" into its components, can be expressed as follows:

Healthy aging risk = (b0) sitting watching TV + (b1) sitting at work + (b2) other sitting at home +(b3) LPA at work +(b4) LPA at home + (b5) MVPA + (b6) sleep + (b7) covariates;

Because "total time" is not included in the partition model, it is therefore not held constant. Therefore, each coefficient for a certain behavior represents the effect of adding, rather than substituting, that behavior, which indicates that time per day is infinite.

© 2024 Shi H et al. *JAMA Network Open.*

Because the proportion of missing values for each exposure was less than 5% (the highest was only 3.1% for sitting at work or away from home or while driving), in the main ISM analysis, imputation using the median values was used. In addition, we performed the complete cases analysis. To further verify the robustness of our results, for women who had at least 1 of the time use variables missing, we used multiple imputations (10 imputations) and the Expectation-Maximisation algorithm [17,18]. The multiple imputation model included age, and non-missing time use variables as covariates.

Similarly, we analyzed the independent and replacement association of all exposures with the four domains of healthy aging. Potential heterogeneity in the relationship was also explored by stratified analysis by MVPA (being physically active, inactive). Participants were dichotomized into physically active and inactive groups using a threshold of 7.5 MET-hours/week, corresponding to the minimum physical activity recommendations [19] (moderate-intensity activity, 3 MET for 0.5 hour on 5 days/week = 3*0.5*5 =7.5 MET-hours/week). The significance of interaction between the stratification variable and exposures was tested using the likelihood ratio test. In secondary analyses, to further evaluate the association between these exposures and healthy aging among survivors, we excluded participants who died before 2012 from usual agers, and then repeated all analyses.

All statistical tests were two-sided and *P* values <0.05 were considered statistically significant. Data management and statistical analyses were performed using SAS 9.4 (SAS Institute, Cary, NC).

eReferences

1. Rowe JW, Kahn RL. Successful aging. *The Gerontologist.* 1997;37(4):433-440.
2. Sun Q, Townsend MK, Okereke OI, Franco OH, Hu FB, Grodstein F. Physical activity at midlife in relation to successful survival in women at age 70 years or older. *Archives of internal medicine.* 2010;170(2):194-201.
3. Ma W, Hagan KA, Heianza Y, Sun Q, Rimm EB, Qi L. Adult height, dietary patterns, and healthy aging. *The American journal of clinical nutrition.* 2017;106(2):589-596.
4. Li S, Hagan K, Grodstein F, VanderWeele TJ. Social integration and healthy aging among U.S. women. *Preventive medicine reports.* 2018;9:144-148.
5. Shi H, Huang T, Ma Y, Eliassen AH, Sun Q, Wang M. Sleep Duration and Snoring at Midlife in Relation to Healthy Aging in Women 70 Years of Age or Older. *Nature and science of sleep.* 2021;13:411-422.
6. Guralnik JM, Kaplan GA. Predictors of healthy aging: prospective evidence from the Alameda County study. *American journal of public health.* 1989;79(6):703-708.
7. Ware JE, Jr., Sherbourne CD. The MOS 36-item short-form health survey (SF-36). I. Conceptual framework and item selection. *Medical care.* 1992;30(6):473-483.
8. de Craen AJ, Heeren TJ, Gussekloo J. Accuracy of the 15-item geriatric depression scale (GDS-15) in a community sample of the oldest old. *International journal of geriatric psychiatry.* 2003;18(1):63-66.
9. Go RC, Duke LW, Harrell LE, et al. Development and validation of a Structured Telephone Interview for Dementia Assessment (STIDA): the NIMH Genetics Initiative. *Journal of geriatric psychiatry and neurology.* 1997;10(4):161-167.
10. Amariglio RE, Townsend MK, Grodstein F, Sperling RA, Rentz DM. Specific subjective memory complaints in older persons may indicate poor cognitive function. *Journal of the American Geriatrics Society.* 2011;59(9):1612-1617.
11. Hu FB, Li TY, Colditz GA, Willett WC, Manson JE. Television watching and other sedentary behaviors in relation to risk of obesity and type 2 diabetes mellitus in women. *Jama.* 2003;289(14):1785-1791.
12. Hu FB, Sigal RJ, Rich-Edwards JW, et al. Walking compared with vigorous physical activity and risk of type 2 diabetes in women: a prospective study. *Jama.* 1999;282(15):1433-1439.
13. Willett WC, Sampson L, Stampfer MJ, et al. Reproducibility and validity of a semiquantitative food frequency questionnaire. *American journal of epidemiology.* 1985;122(1):51-65.
14. Yuan C, Spiegelman D, Rimm EB, et al. Relative Validity of Nutrient Intakes Assessed by Questionnaire, 24-Hour Recalls, and Diet Records as Compared With Urinary Recovery and Plasma Concentration Biomarkers: Findings for Women.

© 2024 Shi H et al. *JAMA Network Open.*

American journal of epidemiology. 2018;187(5):1051-1063.

15. Chiuve SE, Fung TT, Rimm EB, et al. Alternative dietary indices both strongly predict risk of chronic disease. *The Journal of nutrition.* 2012;142(6):1009-1018.
16. Spiegelman D, Hertzmark E, Wand HC. Point and interval estimates of partial population attributable risks in cohort studies: examples and software. *Cancer causes & control : CCC.* 2007;18(5):571-579.
17. Stamatakis E, Rogers K, Ding D, et al. All-cause mortality effects of replacing sedentary time with physical activity and sleeping using an isotemporal substitution model: a prospective study of 201,129 mid-aged and older adults. *The international journal of behavioral nutrition and physical activity.* 2015;12:121.
18. Stamatakis E, Gale J, Bauman A, Ekelund U, Hamer M, Ding D. Sitting Time, Physical Activity, and Risk of Mortality in Adults. *Journal of the American College of Cardiology.* 2019;73(16):2062-2072.
19. Katzmarzyk PT, Church TS, Craig CL, Bouchard C. Sitting time and mortality from all causes, cardiovascular disease, and cancer. *Medicine and science in sports and exercise.* 2009;41(5):998-1005.

© 2024 Shi H et al. *JAMA Network Open.*
