# Supplementary material for: Sedentary Behaviors, Light-Intensity Physical Activity, and Healthy Aging
Source: JAMA Netw Open. 2024 Jun 11;7(6):e2416300. doi: 10.1001/jamanetworkopen.2024.16300 (PMC11167497; doi:10.1001/jamanetworkopen.2024.16300)
Supplement: Supplement 2. — Data Sharing Statement [file jamanetwopen-e2416300-s002.pdf]

Data Sharing Statement

Shi. Sedentary Behaviors, Light-Intensity Physical Activity, and Healthy Aging. *JAMA Netw Open*. Published June 11, 2024. doi:10.1001/jamanetworkopen.2024.16300

Data
Data available: No
